# Delineating the structural, functional and evolutionary relationships of sucrose phosphate synthase gene family II in wheat and related grasses

**DOI:** 10.1186/1471-2229-10-134

**Published:** 2010-06-30

**Authors:** Shailendra Sharma, Nese Sreenivasulu, Vokkaliga Thammegowda Harshavardhan, Christiane Seiler, Shiveta Sharma, Zaynali Nezhad Khalil, Eduard Akhunov, Sunish Kumar Sehgal, Marion S Röder

**Affiliations:** 1 Leibniz Institute of Plant Genetics and Crop Plant Research (IPK), Corrensstr. 3, D-06466 Gatersleben, Germany; 2 Sardar Vallabh Bhai Patel University of Agriculture and Technology, Modipuram, Meerut, Uttar Pradesh 250110, India; 3 Iwate Biotechnology Research Center, Narita 22-174-4, Kitakami, Iwate 024-0003, Japan; 4 Plant Breeding Institute, Christian-Albrechts University of Kiel, Olshausenstrasse 40, 24098 Kiel Germany; 5 Department of Agronomy and Plant Breeding, College of Agriculture, Isfahan University of Technology, 841568311, Isfahan, Iran; 6 Department of Plant Pathology, Throckmorton Plant Sciences Center, Kansas State University, Manhattan, KS 66506, USA

## Abstract

**Background:**

Sucrose phosphate synthase (SPS) is an important component of the plant sucrose biosynthesis pathway. In the monocotyledonous Poaceae, five *SPS *genes have been identified. Here we present a detailed analysis of the wheat *SPSII *family in wheat. A set of homoeologue-specific primers was developed in order to permit both the detection of sequence variation, and the dissection of the individual contribution of each homoeologue to the global expression of *SPSII*.

**Results:**

The expression in bread wheat over the course of development of various sucrose biosynthesis genes monitored on an Affymetrix array showed that the *SPS *genes were regulated over time and space. *SPSII *homoeologue-specific assays were used to show that the three homoeologues contributed differentially to the global expression of *SPSII*. Genetic mapping placed the set of homoeoloci on the short arms of the homoeologous group 3 chromosomes. A resequencing of the A and B genome copies allowed the detection of four haplotypes at each locus. The 3B copy includes an unspliced intron. A comparison of the sequences of the wheat *SPSII *orthologues present in the diploid progenitors einkorn, goatgrass and *Triticum speltoides*, as well as in the more distantly related species barley, rice, sorghum and purple false brome demonstrated that intronic sequence was less well conserved than exonic. Comparative sequence and phylogenetic analysis of *SPSII *gene showed that false purple brome was more similar to *Triticeae *than to rice. Wheat - rice synteny was found to be perturbed at the SPS region.

**Conclusion:**

The homoeologue-specific assays will be suitable to derive associations between SPS functionality and key phenotypic traits. The amplicon sequences derived from the homoeologue-specific primers are informative regarding the evolution of *SPSII *in a polyploid context.

## Background

Sucrose plays an important role in the plant life cycle. As the major photosynthetic product, it is essential for growth, the synthesis of biomass and as a carbon and energy source. In the cereals, it is converted into starch and storage proteins, while many dicotyledonous species use it to form lipids and/or storage proteins [1,2]. Under stressful conditions (e.g, low temperature or drought ), the plant cell typically accumulates sucrose as a protective osomoticum [3,4]. Sucrose phosphate synthase (SPS) is one of the main regulatory enzymes involved in sucrose biosynthesis pathway in wheat and many other crop species [5]. It catalyzes the conversion of Fructose-6-Phosphate and UDP-glucose into Sucrose-6-phosphate, which is subsequently hydrolysed to sucrose by the action of sucrose phosphate phosphatase (SPP).

In important crop plants like maize, rice and sugarcane, plant growth and productivity have been correlated with SPS activity. SPS activity has been correlated with sucrose accumulation in sugarcane stems [6-8], while in maize, correlations have been demonstarted with vigour and biomass yield [9,10]. Genetic studies in maize have also shown that grain yield QTL (quantitative trait loci) are linked to SPS activity QTL, as are ADP-glucose pyrophosphorylase (another starch biosynthesis enzyme) activity QTL [11-13]. In rice, the location of plant height QTL appears to coincide with that of *OsSPS1*, as the transgenic plants with increased SPS activity grew taller than the non-transformed control [14]. In tobaccco lines engineered to over-express SPS, UDP- glucose pyrophosphorylase and sucrose synthase, plant height was increased and flowering time delayed [15], while the heterologous expression of *AtSPS *induced longer stems and greater biomass [16]. Finally, the over-expression of *ZmSPS *in potato was shown to improve a number of yield-realted characters [17], while in tomato, it altered the pattern of carbohydrate partitioning in the leaf [18].

The regulation of SPS activity is rather complex, and involves fine tuning at both the transcriptional and the post-translational level [2]. Among the dicotyledonous plant species, three *SPS *gene families have been recognized, while in the monocotyledonous Poaceae species wheat, maize and rice, there are five gene families [5]. Here, we report the temporal patterns of expression of *SPS *and *SPP*, and the discovery of structural polymorphisms between homoeologous copies of *SPSII*. We have also identified the presence of unspliced introns in one of its homoeologues. Evolutionary relationships among the *SPSII *homoeologues have been illustrated by comparisons between the gene sequences present in hexaploid wheat and its progenitors. Finally, we extend the phylogeny to other grass species.

## Results

### Gene expression analysis of sucrose biosynthesis genes during wheat plant ontogeny

**Figure 1:**
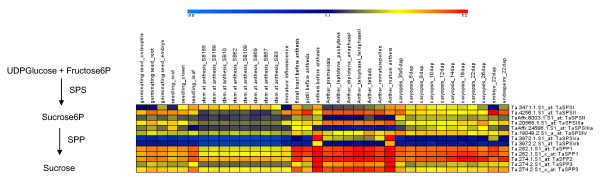
**Spatial and temporal expression patterns of *SPS *and *SPP *during plant development**. The expression values were calculated on a logarithmic scale (base 2). Red signal denotes high expression; yellow, moderate expression and blue, low expression. Affymetrix gene IDs are shown on the right panel, and the developmental stage sampled in vertical columns by the top panel.

As a first step, the wheat Affymetrix probe sets representing *SPS *and *SPP *members were identified, based on the criteria used to define the five SPS [5] and the three *SPP *[19] types. The expression patterns of these gene family members was extracted from the normalized expression data from imbibed embryo, coleoptile, seedling root, crown, seedling leaf, stem (at anthesis), developing anther, developing caryopsis (sampled at 3-5 dap, 8 dap, 18 dap and 22 dap), and the developing endosperm and embryo at 22 dap. Except for *SPPIII*, the *SPP *genes were expressed throughout plant development, although their level tended to be highest in the developing anther and developing caryopsis (Figure 1). Different SPS members show highly regulated spatio-temporal expression patterns during plant ontogeny, suggesting the existence of fine regulation at the first step of sucrose biosynthesis.

As *SPSI *was prominently expressed in the seedling leaf and the mature stem (Figure 1) its function may be associated with the re-synthesis of sucrose from fructan. *SPSII *(Ta.4256.1.S1_at), *SPSIII *(Ta.20568.1.S1_at), *TaSPSIV *and *TaSPSVa *were prominently expressed during microsporogenesis (at premeiosis, throught meiosis and in immature pollen) and in the mature anther. All the *SPP *families were highly expressed during anther development (Figure 1). Thus, these genes are clearly important for normal pollen/anther development. Interestingly, note that in the *Arabidopsis thaliana *mutant *kns2-1*, an altered *SPS *glycosyltransferase I domain disrupts the function of sucrose synthesis, and this leads to changes in the synthesis of the pollen primexine or callose [20]. In the wild type, SPS5.2 (*KNS2*) is expressed in uni- and bicellular microspores as well in more mature anthers. The present data showed that probably all the *SPS *and *SPP *genes were constitutively expressed throughout caryopsis development, indicating that more than one SPS isoforms contributes to overall SPS activity (Figure 1).

Furthermore, it is important to know how these different SPS members are spatially expressed in different seed tissues and possible co-expression of these members in the same cell for making hetero-oligomeric complexes to be functional. The developing caryopsis imports sucrose to driving its growth and allow storage of metabolites. The expression of *SPS *and *SPP *members within the developing caryopsis suggests that sucrose cleavage, transport and re-synthesis during its internal transport can be differentially regulated within various distinct tissues. Of particular interest was the observation that both variants of *SPSII *are differentially expressed in the developing caryopsis. Thus, Ta.4256.1.S1_at was highly expressed both throughout anther development and during late embryo development, while TaAffx.8003.1.S1_at was expressed most strongly during early caryopsis development. The contribution of the individual *SPSII *homeologues and the occurance of alternative splicing are expanded upon in the following section. Besides alternative splicing, a contributory reason for the present exclusive focus on the *SPSII *family was that there were four *SPSII *cDNA sequences available in the public domain, which gave the opportunity to design homoeologue-specific primers for three wheat genomes. Insufficient sequence data are presently available for the other *SPS *families. Therefore, in order to study polyploidization and phylogenetics relationship between three wheat genomes, SPSII was selected as best suited candidate.

### Structural analysis of SPSII genes in wheat and its relatives

**Figure 2:**
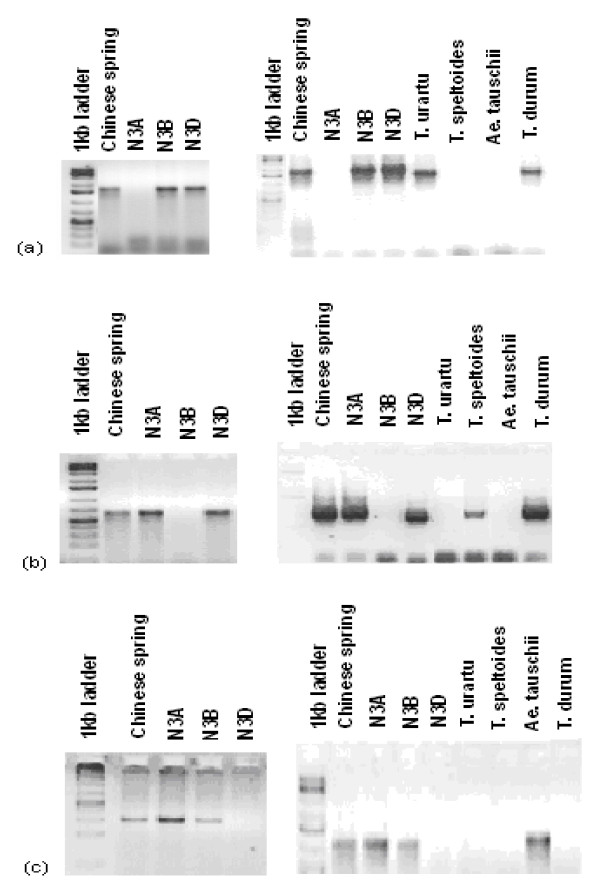
***SPSSII *homoeologue-specific primer driven PCR of cv. 'Chinese Spring', relevant nulli-tetrasomic lines and selected progenitor species**. (a) A genome copy (primer pair w-spsII-dL & w-spsII-24R), (b) B genome copy (primer pair w-spsII-65L & w-spsII-73R), (c) D genome copy (primer pair w-spsII-1DL & ss-spsII-D11R).

Both introns and exons were targeted for the design of homoeologue-specific primers (Figure 2). The resulting amplicons allowed for the sequencing of portions of each of the *SPSII *homoeologues. In all, 7,364 bp of the cv. Chinese Spring A genome homoeologue sequence was acquired from a combination of homoeologue-specific amplicons and flow-sorted chromosome arm 3A sequences (kindly provided by Dr. E. Akhunov). We acquired 3,883 bp of the B genome homoeologue sequence, and 1,184 bp of the D genome homoeologue, both from cv. Chinese spring (Additional file 1). Sequence of the *SPSII *orthologues in *Triticum urartu *(einkorn wheat - genome AA; A genome progenitor), *T. speltoides *(genome SS ~ BB; putative B genome progenitor)*, Aegilops tauschii *(goatgrass-D genome progenitor) and barley (genome HH) was obtained in a similar fashion. Their alignment is provided as Additional file 2. *SPSII *has 12 introns and 13 exons in wheat, barley, rice, sorghum and purple false brome (*Brachypodium distachyon*) (Additional file 1, Figure 3). In rice, sorghum and purple false brome, the gene length is 12,838 bp, 12,456 bp and 8,492 bp, respectively (Additional file 1). In einkorn and *T. speltoides *six exons and six introns were amplified, while in goatgrass, this was extended to eight exons and seven introns. In hexaploid wheat, certain regions could not be sequenced either due to the presence of large introns or due to unavailability of homoeologue-specific primers. The intronic sequences were more polymorphic than the exonic ones, which were highly conserved among the species compared (Additional files 3, 4, 5, 6). In the six exons for which sequence was available, the level of similarity level ranged from 83.1% (between barley and sorghum) to 99% (between the wheat A genome homoeologue and einkorn) (Additional file 6b). In contrast, for the six intronic regions compared, the similarity level ranged from 35.5% (between purple false brome and sorghum) to 99% (between the wheat A genome copy and that of einkorn) (Additional file 6c). Intron 12 of the wheat B genome homoeologue was unspliced, since it was present in the cDNA AF347066 [5]. Sequence alignment and subsequent cluster analysis showed that the purple false brome sequence was more similar to those of wheat and barley in comparison to rice sequence, and the sorghum sequence was the one most highly diverged from that of wheat (Figure 4), as was the expectation on taxonomic grounds [21].

**Figure 3:**
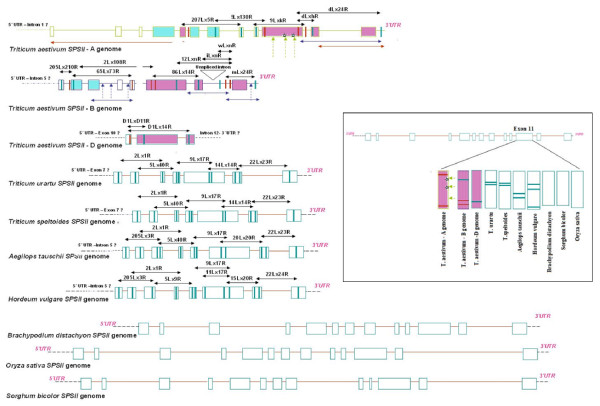
**Structure of *SPSII *genes in wheat and other grasses**. Exons are represented by closed boxes separated by horizontal lines representing introns. Exons filled with horizontal blue lines represent regions containing a glycosyltransferase domain, those with horizontal pink lines contains sucrose phosphate synthase/possible sucrose phosphate phosphatase domains. Vertical red bars show the placement of the homoeologue-specific primers, and vertical blue ones the placement of common primers used in combination with the homoeologue-specific ones. Each homoeologue-specific primer pair is shown above a horizontal two headed arrow depicting the amplicon. The two headed arrow horizontal red line for the A genome copy indicates flow-sorted chromosome arm 3A sequences. The two headed arrow horizontal blue lines represent genetically mapped regions. The location of SNPs is depicted by vertical dashed arrows. Asterisks show the location of non-synonymous SNPs. Box on the right hand side of figure depicts the vertical alignment of eleventh exon, amplified in all 10 genomes, to the reference gene at the top.

**Figure 4:**
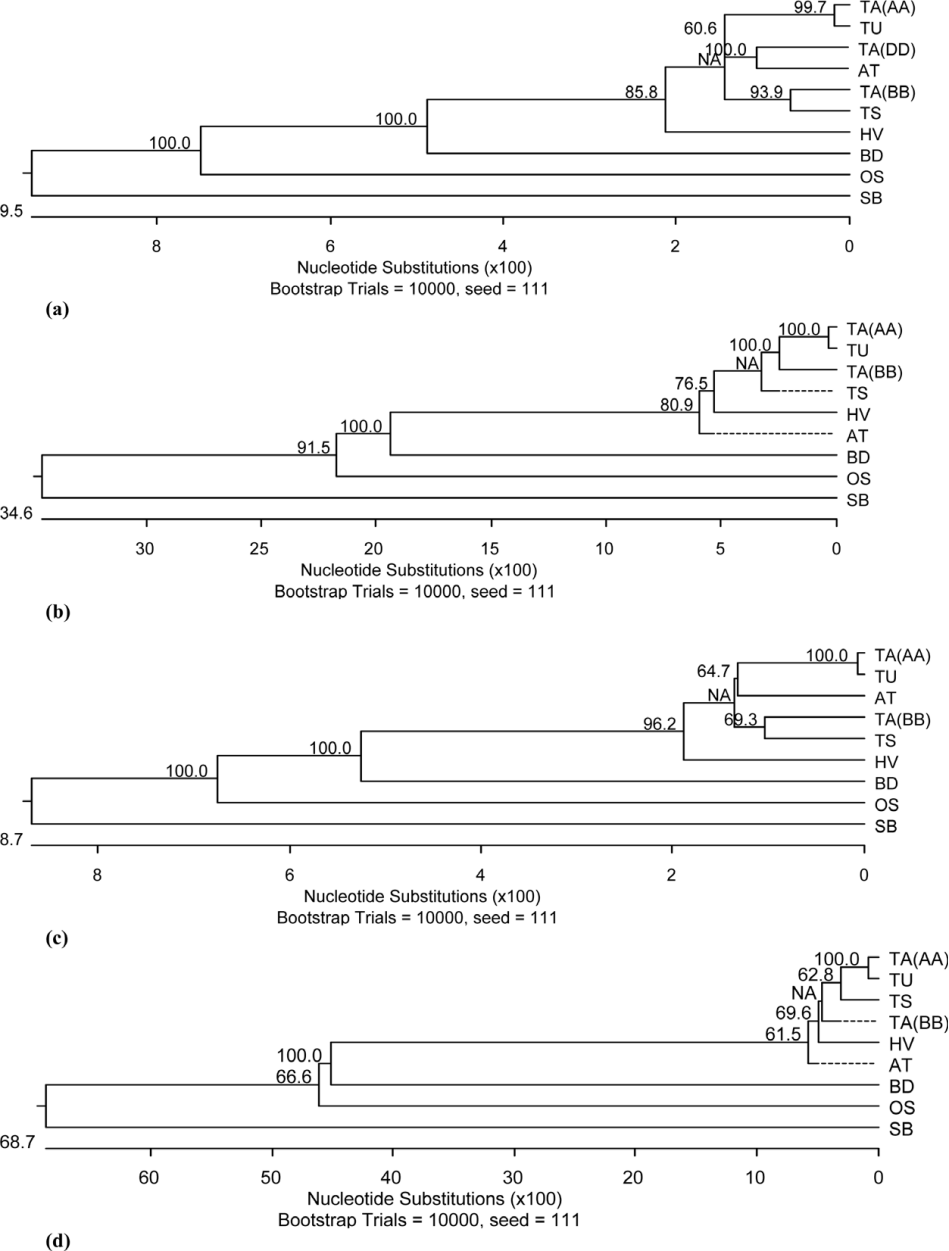
**Phylogeny of *SPSII***. (a) A phylogenetic tree based on the alignment of *SPSII *sequences acquired from ten genomes (see also Fig. 4), (b) A phylogenetic tree based on the alignment of *SPSII *intron 7 to exon 13, based on nine genomes (see also Additional file 2), (c) A phylogenetic tree based on the alignment of *SPSII *exon 8 to exon 13 based on nine genomes (see also Additional file 3), (d) A phylogenetic tree based on the alignment of *SPSII *intron 7 to intron 12 based on nine genomes (See also Additional file 4). TA(AA): *Triticum aestivum *A genome, TA(BB): *Triticum aestivum *B genome, TA(DD): *Triticum aestivum *D genome, TU: *Triticum urartu*, TS: *Triticum speltoides*, AT: *Aegilops tauschii*, HV: *Hordeum vulgare*, OS: *Oryza sativa*, SB: *Sorghum bicolor*, BD: *Brachypodium distachyon*. Dashed lines indicate a negative branch length.

### Genetic mapping of TaSPSII genes in wheat

**Figure 5:**
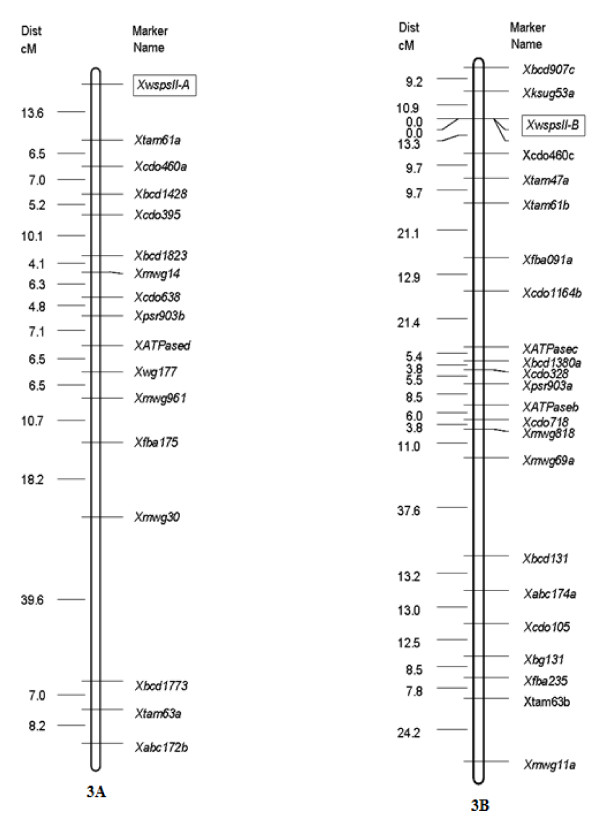
**Genetic maps showing the location of the chromosome 3A and 3B *SPSII *homoeologues**.

The homoeologue-specific primers developed for each of the wheat *SPSII *copies were validated by an analysis of the nulli-tetrasomic lines of cv.'Chinese Spring'; this showed that each amplicon was derived from a member of the homoeologous group three chromosomes (Figure 2). A polymorphism for the A genome copy between the parents of the ITMI (International Triticeae Mapping Initiative) mapping population allowed it to be located 13.6 cM distal of *Xtam61a *in the sub-telomeric of the short arm of chromosome 3A (Figure 5). The B genome copy was placed in a matching location on chromosome 3B between *Xksug53a *and *Xcdo460c *(Figure 5). The D genome copy was not mappable in the ITMI mapping population, but was localised to D genome through anueploid stocks.

### Detection of SNP diversity

The *SPSII *amplicons obtained from 27 diverse wheat accessions were sequenced in a search for single nucleotide polymorphisms (SNPs). For the A genome copy, the intron 6 to exon 12 region was targeted, while for the B genome, the target was exon 6 to the 3' UTR, and, for the D genome copy, intron 10 to exon 12. Three SNPs were uncovered in the 3A gene, all within an exonic region corresponding to the sucrose phosphate synthase/possible sucrose phosphate phosphatase domain. Of the three SNPs, two were non-synonymous SNPs; the first induced the residue change argeneine^1554 ^to lysine^1554^, and the second from alanine^1599 ^to valine^1599 ^(Figure 3, Table 1). Of the four B genome copy SNPs detected, three were located within intronic sequences, and one within the 3' UTR (Figure 3, Table 1). No SNP was detected within the D genome copy. Overall, it was possible to define four distinct haplotypes for each of the A and the B genome copies (Table 1).

**Table 1:** Haplotypes for *SPSII *gene obtained in wheat A and B genomes

Haplotypes -A Genome	SNP1 (Exonic, 1432 bp	SNP2* Exonic, 1554 bp)	SNP3* (Exonic, 1599 bp)	Cultivars per haplotype	Haplotypes-B genome	SNP1 (Intronic, 451 bp)	SNP2 (Intronic, 523 bp)	SNP3, (Intronic 793 bp)	SNP4 (3'UTR)	Cultivars per haplotype
SPShap1	C	G (ARG)	C (ALA)	10	SPShap1	A	T	G	C	15
SPShap2	T	G (ARG)	T (VAL)	9	SPShap2	G	T	A	G	3
SPShap3	T	G (ARG)	C (ALA)	6	SPShap3	G	T	G	G	6
SPShap4	C	A (LYS)	C (ALA)	2	SPShap4	G	C	G	G	3
				Total = 27						Total = 27

Note: Position of the SNPs are based on sequence alignment (supplementary Figure 1). Asterisk showed the location location of non synonymous

SNPs.

### Three genomes differentially contributes to the expression of TaSPSII

**Figure 6:**
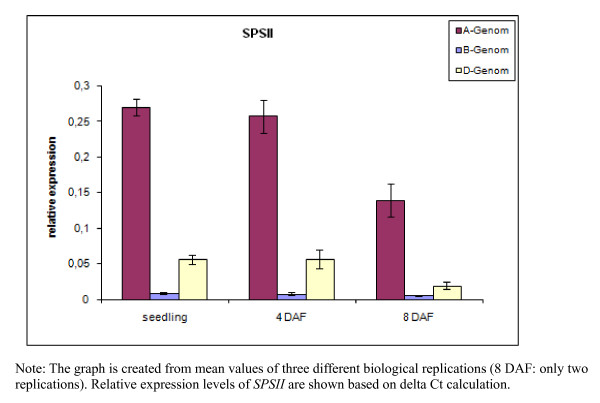
**Relative expression levels of *SPSII *homoeologues in cv 'Prinz'**. Relative expression levels of SPSII shown are based on delta Ct calculation. To compare data from different PCR runs or cDNA samples, C_T _values for all genes were normalized to the C_T _value of the housekeeping gene (serine/threonine protein phosphatase, TaPP2A) included in each PCR run. The expression level of each gene of interest (GOI) is presented as 2^-ΔCT^, where ΔC_T _= C_TGOI _- C_TREF_. Mean values were assessed from three biological replicates for seedling materials and caryopses sampled four days after anthesis, and from two replicates for caryopses sampled eight days after anthesis. Error bars indicate standard deviations from the mean.

Quantitative real time PCR based on exonic sequences of the A and D genome copies, and within the unspliced intronic region of the B genome copy was applied to various tissues of, cv 'Prinz'. *SPSII *was expressed during germination as well as in the developing caryopsis at four and eight days after anthesis (DAF). A similar level of expression was measured in the germinating seed and in the developing caryopsis at four DAF, and this fell somewhat by eight DAF. The A genome copy dominated the global expression of *SPSII*; there was very little contribution from the B genome copy (Figure 6).

## Discussion

### Genomic sequence analysis of SPSII genes

Sequence variation in sucrose biosynthesis pathway genes such as *SPS *is of particular interest because its consequence can be a change at the level of phenotype, sometimes involving vigour, biomass productivity and economic yield. Markers from the expressed portion of genome, that are responsible for some important physiological aspects, have been developed in wheat [22-26]. A particular complication in bread wheat is that most genes which are present in single copy in the diploid grasses such as rice and purple false brome are represented by three copies. In order to interpret wheat gene expression data, it is therefore necessary to derive specific assays for each homoeologous copy, as we have described here for *SPSII *(Table 2). These assays are also necessary for the application of pyrosequencing as a SNP discovery platform [27,8].

**Table 2:** Primer sequences used for the amplification of *SPSII *gene in wheat and other related grasses

Forward primer (5'-3')	Reverseprimer (5'-3')	PCR profile
**A. *Triticum aestivum*- A genome**		**1**
1. w-spsII-207L* (*CGTTTTATCCCCCCATAAGAGA*)	w-spsII-5R (CCCAAATGGTTCAATATAAGCACA)	
2. w-spsII-5L (AGCACAAATGGAGCTGTTTTG)	w-spsII-130R* (AATATCCCTCAAAGAGTCACCAC)	
3. w-spsII-9L (GCTTATGGTCTACCTATGGTTGCT)	w-spsII-kR* (ATAGTAGGGAAGGTTGAGAAAATCA)	
4^#^. w-spsII-dL* (AGAAAAGAATAGCGAGAGTGGAA)	w-spsII-24R (AGATGCTAATTATTCGTAGAGATGCAT)	
5^a^.w-spsII-dL (AGAAAAGAATAGCGAGAGTGGAA)	w-spsII-hR (ATGCTTGAATTCTCATTGTCTTC)	
**B. *Triticum aestivum *-B genome**		**1**
1. w-spsII-205L (AGCTGCTACGGTCGTGAAATG)	w-spsII-210R* (CTCTTATGGGGGGATAAAAAT)	
2^#^. w-spsII-65L (CATTTGGTGAACACCATGAGCTA)	w-spsII-73R* ( CCGGTGTATATCGACAGGT)	
3. w-spsII-2L (GCTGGTTAAGGCATTTGGT)	w-spsII-108R* (AACTCCTTCCATATTTCGACATGT)	
4. w-spsII-86L* (GAAAAATCAGGCAACATGTCG)	w-spII-14R (TGCTTGGGATCGTGATGCT)	
5. w-spsII-iL (CACTAAGGTTTGGTTATTCCTATAACTGT)	w-spsII-nR * (CCTGCACAAAGATGAACAAACG)	
6^#^. w-spsII-mL* (AACCACAAAGTCCTGATGGTACG)	w-spsII-24R (AGATGCTAATTATTCGTAGAGATGCAT)	
7^#^. w-spsII-12L (AAACTATTTGTCACGGGTTGGT)	w-spsII-nR (CCTGCACAAAGATGAACAAACG)	
8^a^.w-spsII-wL (GTCGTTTGACACCTCATTGTTG)	w-spsII-nR (CCTGCACAAAGATGAACAAACG)	
**C. *Triticum aestivum *-D genome**		**2**
1 w-spsII-D1L* (CCAAAATGGTGGGCCTGTTG)	w-spsII-14R (TGCTTGGGATCGTGATGCT)	
2^a^. w-spsII-D1L (CCAAAATGGTGGGCCTGTTG)	w-spsII-D11R GAAACTTCAGTAGCATCATCACTCTTTG)	
**D. *Triticum urartu*, *Triticum speltoides*, *Aegilops tauschii*, *Hordeum vulgare***		**1**
1. w-spsII-205L (AGCTGCTACGGTCGTGAAATG)	w-spsII-3R (TGGAACTTCAGATTGCTTATGG)	
2. w-spsII-2L (GCTGGTTAAGGCATTTGGT)	w-spsII-1R (GGTGAGCCCAAATGGTTCAATATA)	
3. w-spsII-5L (AGCACAAATGGAGCTGTTTTG)	w-spsII-40R (CCCGGTGTATATCGACAGGC)	
4. w-spsII-9L (GCTTATGGTCTACCTATGGTTGCT)	w-spsII-17R (GATGTTGACAATACAAAACCAAGAGC)	
5. w-spsII-22L (TTCATACCTGTTCTAGCATCACGA)	w-spsII-23R (GGAACCTATGAACAATTTCACCA)	
6. w-spsII-14L (GAGCAGCATCAGAGATACATCCTT)	w-spsII-14R (TGCTTGGGATCGTGATGCT)	
7. w-spsII-20L (AAAGCTGGGGCCACCAC)	w-spsII-20R (TCGTGATGCTAGAACAGGTATGAA)	
8. w-spsII-22L (TTCATACCTGTTCTAGCATCACGA)	w-spsII-24R (AGATGCTAATTATTCGTAGAGATGCAT)	
9. w-spsII-22L (TTCATACCTGTTCTAGCATCACGA)	w-spsII-31R (ACCGTCATGTTCGACAGCTCTAC)	
10. w-spsII-11L (AATCAAAATGATATAGCTGAGGCACTT)	w-spsII-17R (GATGTTGACAATACAAAACCAAGAGC)	
11. w-spsII-15L (GTTAACATCTGGGGGCATAGAAAT)	w-spsII-20R (TCGTGATGCTAGAACAGGTATGAA)	
12. w-spsII-5L (AGCACAAATGGAGCTGTTTTG)	w-spsII-9R (CACCATTTTGGGTAGCAACC)	

*represents homoeologue-specific primers ; ^a^represents primer pairs used for quantitative real time PCR; ^# ^represents primer pairs used for genetic mapping. For details of PCR profile please see methods section

### Frequency of SNPs

The low level of SNP within *SPSII *suggests that variation in its sequence is constrained by selection against changes in its physiological activity. Other starch metabolism enzyme encoding genes have similarly been associated with little sequence variation [22,28]. The observed SNP frequency was 1 per 797 bp within the A genome copy, and 1 per 916 bp within the B genome copy, frequencies which are typical for bread wheat [29-31]. Most of the *SPSII *SNPs were located within either an intron or the 3' UTR, with only two exonic non-synonymous ones uncovered. These latter substitutions may be of physiological significance, given that an alanine to valine change compromised the processing of a 22 kDa protein in maize [32], and that an arginine to lysine substitution in the bZIP domain of the maize opaque gene abolished the specificity of its DNA binding [33].

### Presence of an unspliced intron in TaSPSII cDNA is mapped to B genome

**Figure 7:**
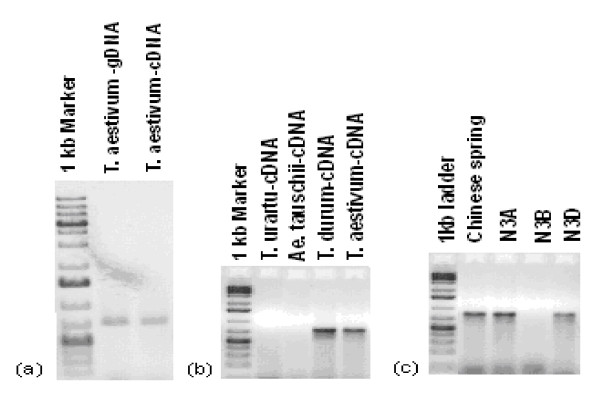
**Amplicon profiling using primer pair w-SPSII-iL & w-SPSII-nR**. (a) gDNA and cDNA of bread wheat, (b) wheat progenitors and bread wheat cDNA, (c) homoeologue specificity (3B copy). N3A, N3B and N3D: nulli-tetrasomic lines lacking, respectively, chromosomes 3A, 3B and 3D.

Intronic sequence was relatively variable at the inter-specific and inter-generic level (Additional files 4, 5c), while exonic sequence was well conserved (Additional files 3, 5a and 5b). This pattern is characteristic of gene sequences in the grasses [21,34]. Analysis of the sequence of *SPSII *cDNA clone AF347066 has demonstrated alternative splicing for one of the *SPSII *genes [5]. The development of homoeologue-specific assays has allowed the unspliced intron to be located to the B genome copy of *SPSII*. Both of the alternative transcripts (spliced and unspliced) occurred in the pre anthesis spike [5]. cDNA of einkorn wheat (genome AA), goatgrass (genome DD), durum wheat (*T. durum*, genome constitution AABB) and bread wheat (genome AABBDD) were amplified using primer pairs w-spsII-iL & w-spsII-nR, which target the unspliced intron in the B genome copy of *SPSII*. Amplification was observed from bread and durum wheat template, but not from either einkorn or goatgrass (Figure 7b). An aneuploid analysis confirmed the B genome specificity of the unspliced intron (Figure 7c). Thus the unspliced intron is clearly restricted to the B genome copy of *SPSII*.

### Contribution of A and D genome for the expression of TaSPSII

All five *SPS *members appeared to be both spatially and temporally regulated. Of special interest in SPSII with two different variants, Ta.4256.1.S1_at: highly expressed throughout anther development as well also during late seed development in embryo and TaAffx.8003.1.S1_at: expressed mainly during early seed development. The expression pattern of Ta.4256.1.S1_at was consistent with that previously exposed by Northern blot analysis [5], but the expression of the latter gene has not been documented before. Although microarray-based expression data reflect very clearly any differences between distinct genes, they are not informative at the level of homoeologues, because the array features are not homoeologue-specific. In this case, homoeologue specific primers could play a vital role in knowing the contribution of three different genomes in terms of gene expression pattern. Similar strategy was also used earlier for studying expression patterns of benzoxazinones biosynthesis in wheat contributed by different genomes [35]. The present data have shown that the A genome copy of *SPSII *dominated the global expression of *SPSII *and that the B genome copy - despite its two alternative transcripts - the least.

### Synteny to rice is perturbed at SPSII locus

At the cDNA sequence level, *SPSII *is highly similar to its rice orthologue, which is located on chromosome 8 [5]. Gene order is largely conserved among the major cereal genomes [36-38], which has allowed for the identification of a syntenic relationship between rice chromosome 8 and the wheat chromosomes of homoeologous group seven. The prediction is therefore that wheat *SPSII *should map to wheat chromosomes 7A, 7B and 7D, and not, as is the case, to chromosomes 3A, 3B and 3D. The chromosomal location that we have deduced is secure, as it relies on a combination of anaeuploid analysis, genetic mapping, the placement of *SPSII *ESTs http://wheat.pw.usda.gov/cgi-bin/westsql/map_locus.cgi, and the presence of *SPSII *sequence on flow-sorted chromosome 3A template. Rice chromosome 1 has been reported to be highly syntenic to wheat chromosome three [38,39]. In the present study, it appears that a segment at least of the group 3 chromosomes has lost synteny with rice chromosome 1. Failure of synteny and breakdown of colinearity is well documented [24,38,40,41]. Prominent examples involve the regions around both *GBSS1 *[42] and the *Pbf *orthologues in wheat and barley [24]. Of 537 mapped ESTs mapping to the wheat group 3 chromosomes, ~15% shared homology with rice genes mapping outside of chromosome 1, and various complexities associated within co-linear regions suggests that the relationship between these chromosomes is not straightforward [38]. Similarly, the analysis of 1 Gb of chromosome 3B has identified the presence of gene sequences whose rice orthologues do not map to chromosome 1 [43], while the annotation of 17 Mb of various regions of chromosome 3B has shown that only half of the predicted genes present had orthologues on rice chromosome 1 [44]. The *SPSII *genes lie in the distal region of their respective wheat chromosome, which suggests that co-linearity with rice may be lost there. Complex duplications, rearrangements and polyploidization events have led to the erosion of synteny and colinearity between rice and wheat [45-47]. The imminent completion of the sequence of the entire chromosome 3B will allow the formulation of a better picture of orthologous relationships between wheat, rice, sorghum and false purple brome, as the genomes of later three species have already been fully sequenced.

### Phylogenetic relationship of TaSPSII in the context of polyploidization among hexaploid wheat and its progeniotors

The sequence alignments and subsequent phylogenetic analyses was carried out considered either exons or introns or both (Figure 4, Additional files 2, 3, 4, 5, 6). The hexaploid wheat A genome copy consistently appeared closest to that of einkorn wheat, the A genome progenitor. At the level of exons only, the B genome copy appeared closely related to that of *T. speltoides*, a species considered to be taxonomically close to the B genome progenitor. Branching patterm in dendrogram was little disturbed when considered only introns depicting nonconserved nature of introns. With respect to the D genome copy, the comparison with its goatgrass orthologue was only possible on the basis of the sequence of one large exon. This showed that the D genome copy was more similar to the goatgrass orthologue than to any of the others. The level of homology in the exonic sequence between the A genome and the einkorn copy was 99.9%, decreasing to 99.0% when intronic sequences were compared (Additional file 6). The equivalent similarities between the B genome and the *T. speltoides *copies were 97.9% and 95.1% (Additional file 6). and that between the D genome and the goatgrass exonic sequence was 98.0%. Thus, polyploidization has left exonic sequence largely unchanged, while intronic sequence has been able to drift somewhat, as has been repeatedly observed in such comparisons [22,34]. The origin of bread wheat has involved two separate allopolyploidization events, the first between einkorn and a species closely related to *T. speltoides*, and the second between the resulting wild tetraploid *T. dicoccoides *and goatgrass [48]. The phylogeny predicted from the sequences of the *SPSII *homoeologues and wild species orthologues is fully consistent with this settled origin (Figure 4) and supports the previous published studies [49,50].

### Phylogenetic relationship of TaSPSII between different grasses

The phylogenetic analysis showed that the purple false brome *SPSII *sequence is more similar to those of wheat and barley than to that of rice, not only in terms of the sequence itself, but in terms of the gene's overall length. The purple false brome *SPSII *fragment extending from exon 8 to the 3'end of the gene (a region available for nine of the ten sequences) was 518 bp longer than the longest wheat or barley sequence, while this difference was 3,199 bp in rice and 3,663 bp in sorghum. A number of phylogenetic studies have shown that the taxonomic separation between the *Triticeae *and purple false brome is much less than between them and rice [51-53], which is the reason why purple false brome has been so readily adopted as a model genome for the *Triticeae *[53-55].

## Conclusion

A study of the expression of sucrose biosynthesis gene family members has revealed that *SPS *genes are highly regulated both spatially and temporally. We have been able to locate the *SPSII *homoeoloci to the short arms of their respective group 3 chromosomes, and have shown that their divergence is consistent with the known evolutionary relationships between the component genomes of hexaploid wheat. A phylogenetic study confirmed that purple false brome is a more appropriate model for the *Triticeae *than rice. We have provided evidence that the syntenic relationship between rice chromosome 8 and wheat group 3 chromosomes is perturbed in the region where the *SPSII *genes map. We have shown that the transcript of the B genome copy of *SPSII *exists as two alternative forms, one with, and the other without an intron. The B genome copy contributed least to the global expression of *SPSII *expression. The development of homoeologue-specific markers for, and SNPs within the wheat *SPSII *genes will enable the definition of any associations between *SPSII *genotype and plant phenotype in terms of vigour, biomass productivity or source-sink relationships.

## Methods

### Plant materials and DNA isolation

Seed stocks of 27 bread wheat accessions and representative progenitors species were obtained from the gene bank of the Leibniz Institute of Plant Genetics and Crop Plant Research, Gatersleben (Table 3). A set of 21 nulli-tetrasomic lines in cv. 'chinese spring' originally obtained from late Dr. E.R. Sears, Coloumbia, MO, was used for chromosome arm assignment of PCR products. Total genomic DNA was extracted from 4-6 g young leaf material as described earlier [56] to be used as a template for PCR amplification.

**Table 3:** Details of wheat accessions used for sequencing

S. No.	Accessions	Country of origin
1	Thatcher (TRI 1308)	USA
2	Maris Huntsman (TRI 10287)	UK
3	TRI 5714	Iran
4	TRI 5869	Iran
5	TRI 8392	Mongolia
6	TRI 9352	Bulgaria
7	TRI 16330	Ethopia
8	TRI 10470	Afghanistan
9	TRI 8161	Egypt
10	Chinese spring (TRI 23666)	China
11	W7984 (mapping parent)	- (synthetic line)
12	Prinz	Germany
13	TRI 2419	Tibet
14	TRI 2425	Tibet
15	TRI 8373	Syria
16	TRI 8392	Mongolia
17	TRI 10330	Kazakhstan
18	TRI 10495	Afghanistan
19	TRI 10866	Nepal
20	TRI 1664	Greece
21	TRI 1706	Albania
22	TRI 1766	Albania
23	TRI 1772	Greece
24	TRI 2421	Tibet
25	TRI 11528	Iraq
26	TRI 11571	Pakistan
27	TRI 1624	Peloponnesia
28	*Triticum urartu *(AA) (TRI 17140)	Lebanon
29	*Aegilops speltoides *(SS BB) (AE 413 )	Israel
30	*Aegilops *tauschii (DD) (TRI 145/96)	Azerbaijan
31	*Triticum dicoccoides *(AABB) (TRI 18495)	Israel
32	*Triticum durum *(AABB) (TRI 19197)	Turkey

### Expression analysis of SPS and SPP members

ESTs/cDNAs sequences for each of the five *SPS *gene families and three different *SPP *gene families in wheat were blasted against the probesets available on the wheat 61 K microarray platform on which corresponding wheat *SPSI *to *SPSV *and *SPP1 *to *SPP3 *cDNA gene sequences were identified with a significant e value of 10^-10 ^or higher. To assess gene expression patterns of *SPS *and *SPP *gene family members during wheat plant development we downloaded CEL files of Affymetrix chip from the publicly available reference experiments (Series GSE12508; GSE9767; GSE6027) covering various tissues during germination (coleoptile, root, embryo), seedling stage (crown, leaf), stem at anthesis, reproductive tissues (immature inflorescence, floral bracts, pistil, mature anthers), various developmental stages of microsporogenesis and seed development. The transcriptome data of 61 K genes was subjected to RMA normalization with linear model using limma package. The log2 expression values were derived, differential expression is calculated with P value correction mode of Benzamini Hochberg and heat maps were generated for the differentially expressed *SPS *and *SPP *gene family members covering the complete wheat plant ontogeny using Genesis software [57].

### The designing of SPSII family specific primers and subsequent PCR and cloning

The cDNA sequences of each *SPS *gene was retrieved from GenBank (accessions AF347064, AF347065, AF347066, AF347067, AF347068, AF347069, AF354298, AF534907 and AY4257109) and aligned using the Multalin [58] program. The *SPSII *gene family is represented by AF347064, AF347065, AF347066 and AF354298. Stretches of sequences limited to a particular gene family formed the basis for designing family specific primers, and in particular for *SPSII *(Table 2). To test the specificity of these family specific primers, the sequence of the resulting amplicons was used as a BLAST query against the GenBank sequence database http://blast.ncbi.nlm.nih.gov/Blast, applying high stringent conditions. Template DNA for the subsequent PCR was represented by cvs. 'Chinese Spring', 'M6' (a synthetic hexaploid wheat) and 'Opata'. Each 25 μl PCR comprised 50-100 ng template, 2.5 μl of buffer containing 1.5 mM MgCl2, 0.2 mM dNTP, 10 μM of each primer and 1 U Taq polymerase. The amplification conditions consisted of an initial denaturation step (94°C/4 min), followed by four cycles of 94°C/1 min, Ta/1 min, 72°C/2 min (where Ta began at 62°C and was reduced by 1°C per cycle), then 30 cycles of 94°C/1 min, 58°C/1 min, 72°C/2 min and completed by a final extension step of 72°C/5 min. This profile is referred to as "PCR profile 1". Amplicons were cloned into the pDrive cloning vector (QIAGEN, Hilden Germany). Plasmids were recovered from 20 clones from each of the three donor varieties using the QIAGEN plasmid purification kit (QIAGEN, Hilden Germany), and these were subjected to sequencing from both ends using a BigDye terminator v3.1 ready reaction cycle sequencing kit in combination with an ABI3730x1 sequencer (Applied Biosystems).

### Phred-Phrap-Consed analysis of sequenced clones

Phred-Phrap-Consed analysis http://www.phrap.org/phredphrapconsed.html was applied to ensure the quality of the sequences taken forward [59,60]. SNPs were detected by polyPhred [61] and visual inspection of Phrap assemblies. Two high quality contigs, assumed to represent two of the predicted three wheat homoeologues, were aligned using Sequencher software (Gene Codes Corporation, Ann Arbor, Michigan), and their consensus sequence formed a megablast [62,63] query against the GenBank database. The outcome of this search was a confirmation that these two sequences were homologous to SPSII and not to any of the other SPS genes. Primer walking was employed to extend the sequence in both directions beyond the cloned region, and the sequence thereby acquired was subjected to megablast to ensure that it was *SPSII *specific.

### Gene annotation, designing of homoeologue-specific primers, sequencing

A provisional gene structure was obtained by aligning the cDNA sequences with the rice genome sequence, and this was later confirmed by direct alignment with wheat genomic sequence. These alignments was performed using Genseqer http://www.plantgdb.org/PlantGDB-cgi/GeneSeqer/PlantGDBgs.cgi software, applying the the rice specific splice site model and a high stringency [64]. Exon anchored primers were designed to span one or more intron using Primer3 software [65]. Homoeologue-specific primers targeted indels and/or base substitutions mainly in the intronic sequence (Table 2), and their specificity was tested by the amplification of DNA from nulli-tetrasomic template. Four primer pairs specific for the 3A copy of SPSII were obtained, and six for the 3B copy (Table 2, Figure 2). However, no 3D copy primers were identified in this way. To obtain an assay for the 3D copy, primers designed to amplify an exonic fragment from bread wheat were applied to the goatgrass accession TRI 145/96, and a region of this amplicon which was specific to goatgrass (839-880 bp, see Additional file 3) was then targeted for successful primer design (Table 2, Figure 2). The A and B genome copies were amplified using PCR profile 1, while this was modified for the D genome copy to an initial denaturation step (94°C/4 min), followed by 35 cycles of 94°C/1 min, 65°C/1 min, 72°C/2 min and completed by a final extension step of 72°C/5 min (PCR profile 2). The primer sets were applied to obtain orthologous sequence from einkorn, *T. speltoides*, goatgrass and barley. For einkorn and *T. speltoides *it was possible to derive the sequence from intron 7 to the 3'end of the gene in this way, and from goatgrass and barley from exon 6 to the 3'end of the gene. For rice, sorghum and false purple brome, sequence information was retrieved from, respectively, TIGR http://rice.plantbiology.msu.edu/, Phytozome http://www.phytozome.net and BrachyBase http://www.brachypodium.org/. All sequences are shown in Additional file 1, and have been deposited in GenBank as accession numbers GU797178, GU797179, GU797180, GU797181, GU797182, GU797183 and GU929217.

### Mapping of homeologue specific amplicons

Homeologue specific markers showing polymorphism between parents of International Triticeae Map ping Initiative (ITMI) population were used for genetic mapping. For genetic mapping 112 recombinant inbred (RI) lines from the ITMI population were used. This population was derived from the cross between W-7984, an amphi hexaploid wheat and Mexican wheat variety Opata 85 from CIMMYT (Centro Internacional de Me joramiento de Maizy Trigo) [66]. The *SPSII *markers were integrated into the framework map composed of RFLP markers using software package MAPMAKER/Exp version 3.0b [67]. Recombination fractions were converted to centimorgans with the Kosambi mapping function [68].

### SNP identification, synteny study and cluster analysis

Primer pairs which produced a locus-specific amplicon were used amplify template DNA from 27 wheat cultivars, using the appropriate PCR profiles described above. The PCR products were purified with the MinElute™ UF PCR purification kit (QIAGEN, Hilden Germany) following the manufacturer's instructions, and subjected to sequencing from both ends. SNPs were identified by Sequencher v4.06 software (Gene Codes Cooperation, Ann Arbor, MI, USA). Position of the various SNPs shown in Additional file 3. Sequence alignment and phylogeny construction were performed using the MegAlign module of DNASTAR Lasergene^® ^v8.0. software. The resulting sequence alignments and phylogenetic trees are shown in Additional files 2, 3, 4, 5 and Figure 4. Phylogenetic trees were subjected to bootstrap analysis, based on 10,000 replicates.

### RNA isolation, cDNA synthesis and qRT-PCR

Total RNA was extracted from cv. 'Prinz' seedlings and developing caryopses at 4, 8 and 12 days after anthesis, and from seedlings of cv. 'Chinese Spring', einkorn, goatgrass and *T.durum *using the TRIzol reagent (Invitrogen GmbH, Karlsruhe, Germany) and RNAeasy columns (Qiagen, Hilden, Germany), as prescribed in the manufacturers' protocols, followed by in-column DNAse digestion. RNA concentration was measured using a NanoDrop photometer (Peqlab), following the manufacturer's instructions. A 2 μg sample of RNA was used as template for cDNA synthesis, using the SuperScript™III kit (Invitrogen GmbH), according to the manufacturer's instructions. cDNA yield was tested by quantitative PCR employing a serine/threonine protein phosphatase gene (*TaPP2A*) as reference. The cDNA was diluted to so that the concentration of all samples lay between ± 1 C_T_. A qRT-PCR assay of the A and D genome *SPSII *homoeologue targeted exonic regions, following recommended guidelines [69], while the B genome homoeologue targeted the unspliced intron. PCRs were performed in optical 384-well plates using the ABI PRISM^® ^7900 HT Sequence Detection System (Applied Biosystems, Foster City, CA, USA) and SYBR^® ^Green to monitor dsDNA synthesis. Each 10 μl reaction contained 5 μl 2 × Power SYBR^® ^Green Master mix reagent (Applied Biosystems), 1 μl cDNA and 200 nm of each homoeologue-specific primer. The PCR profile was 50°C/2 min and 95°C/10 min, followed by 45 cycles of 95°C/15 s, 60°C/60 s. Melting curves were recorded after cycle 45 by heating from 60°C to 95°C at 1.9°C min^-1^. Data were analysed using SDS2.2.1 software (Applied Biosystems). To generate a baseline-subtracted plot of the logarithmic increase in fluorescence signal (ΔR_n_) versus cycle number, the baseline data were collected between cycles 3 and 15. To obtain C_T _values, the amplification plots were analysed at an R_n _threshold of 0.2. To compare data from different cDNA samples, the *SPSII *C_T _values were normalized against the expression of *TaPP2A*. PCR efficiency (E) was estimated from the exponential phase of each amplification, following [70]. Since these lay between 1.8 and 2.0 for all samples, the expression level was calculated as 2^-ΔCT^, where ΔC_T _represented the difference between the C_T _of the *SPSSII *homoeologue and that of *TaPP2A*.

## Authors' contributions

SS carried out the molecular genetic studies, sequence alignment, phylogenetic analysis, splice alignment and primer designing. He conceived the study, participated in its design and drafted the manuscript. NS performed gene expression analysis and drafted part of the manuscript. VTH and CS contributed to cDNA synthesis and qRT-PCR analysis using homoeologue-specific primers. SS and ZNK helped in carrying out some genetic experiments. EA and SKS provided the 3A specific sequence information. MSR coordinated the study, contributed to its conception and design, to interpretation of data and to revising the manuscript critically. All authors read and approved the final manuscript.

## Supplementary Material

Additional file 1 **Intron-exon lengths of *SPSII *gene family determined in 10 different plant genomes**. Click here for file

Additional file 2 **Sequence alignment of *SPSII *gene studied in all 10 genomes using MegAlign (ClustalW, slow/accurate)**. Boxes represented residues different from the consensus. TA(AA): *Triticum aestivum *A genome, TA(BB): *Triticum aestivum *B genome, TA(DD): *Triticum aestivum *D genome, TU: *Triticum urartu*, TS: *Triticum speltoides*, AT: *Aegilops tauschii*, HV: *Hordeum vulgare*, OS: *Oryza sativa*, SB: *Sorghum bicolor*, BD: *Brachypodium distachyon*. Click here for file

Additional file 3 **Sequence alignment of *SPSII *gene region containing six exons and six introns (intron 7 to exon 13) studied in nine genomes (without *Triticum aestivum *D genome)**. TA(AA): *Triticum aestivum-*A genome, TA(BB): *Triticum aestivum-*B genome, AT: *Aegilops tauschii*, HV: *Hordeum vulgare*, TU: *Triticum urartu*, TS: *Triticum speltoides*, BD: *Brachypodium distachyon*, SB: *Sorghum bicolor*, OS: *Oryza sativa*. Sequences from 5' UTR and 3' end are not shown due to unavailbilty of all sequeneces in some cases. Positions with SNP represented by grey boxes. Sequence in red font represented homoeologue-specific primers with 3' end SNP. Sequence in red font with underline represented overlapping forward and reverse homoeologue-specific primers with 3' end SNP. Unspliced intron in TA (BB) sequence is shown by bold italic font. TA(AA): *Triticum aestivum *A genome, TA(BB): *Triticum aestivum *B genome, TU: *Triticum urartu, TS: Triticum speltoides, AT: Aegilops tauschii, HV: Hordeum vulgare, OS: Oryza sativa, SB: Sorghum bicolor, BD: Brachypodium distachyon.* Click here for file

Additional file 5 **Sequence alignment of six introns (intron 7-12) of *SPSII *gene that could be studied in nine genomes (without *Triticum aestivum *D genome) using MegAlign (ClustalW, slow/accurate)**. Boxes represented residues different from the consensus. TA(AA): *Triticum aestivum *A genome, TA(BB): *Triticum aestivum *B genome, TU: *Triticum urartu*, TS: *Triticum speltoides*, AT: *Aegilops tauschii*, HV: *Hordeum vulgare*, OS: *Oryza sativa*, SB: *Sorghum bicolor*, BD: *Brachypodium distachyon*. Click here for file

Additional file 6 **Sequence similarity and divergence of *SPSII *gene based on sequence alignment of different regions (a) Sequence similarity and divergence of *SPSII *gene based on region studied in all ten genomes (including *Triticum aestivum *D genome)**. (b) Sequence similarity and divergence of *SPSII *gene based on six exons (exon 8-13) compared in nine genomes (without *Triticum aestivum *D genome). (c) Sequence similarity and divergence of *SPSII *gene based on six introns (intron 7-12) compared in nine genomes (without *Triticum aestivum *D genome). TA(AA): *Triticum aestivum *A genome, TA(BB): *Triticum aestivum *B genome, TU: *Triticum urartu*, TS: *Triticum speltoides*, AT: *Aegilops tauschii*, HV: *Hordeum vulgare*, OS: *Oryza sativa*, SB: *Sorghum bicolor*, BD: *Brachypodium distachyon*. Click here for file

Additional file 4 **Sequence alignment of six exons (exon 8-13) of *SPSII *gene studied in nine genomes (without *Triticum aestivum *D genome) using MegAlign (ClustalW, slow/accurate)**. Boxes represented residues different from the consensus. TA(AA): *Triticum aestivum *A genome, TA(BB): *Triticum aestivum *B genome, TU: *Triticum urartu*, TS: *Triticum speltoides*, AT: *Aegilops tauschii*, HV: *Hordeum vulgare*, OS: *Oryza sativa*, SB: *Sorghum bicolor*, BD: *Brachypodium distachyon*. Click here for file
