# Supplementary material for: Delineating the structural, functional and evolutionary relationships of sucrose phosphate synthase gene family II in wheat and related grasses
Source: BMC Plant Biol. 2010 Jun 30;10:134. doi: 10.1186/1471-2229-10-134 (PMC3017794; doi:10.1186/1471-2229-10-134)
Supplement: Additional file 1 — Intron-exon lengths of SPSII gene family determined in 10 different plant genomes. [file 1471-2229-10-134-S1.DOC]

**Additional file 1**

**Intron-exon lengths of *SPSII* gene family determined in 10 different plant genomes**

| Exon/Intron/UTR | *Triticum aestivum* (Chinese spring)  (A genome) | *Triticum aestivum* (Chinese spring)  (B genome) | *Triticum aestivum* (Chinese spring)  (D genome) | *Triticum*  *urartu*  (TRI 17140) | *Triticum speltoides*  (AE 413) | *Aegilops tauschii*  (TRI 145/96) | *Hordeum vulgare*  (Morex) | *Oryza sativa*  LOC_Os08g20660 | *Sorghum bicolour*  Sb09g028570.1 | *Brachypodium*  *distachyon*  Bradi3g20120.1 |
| --- | --- | --- | --- | --- | --- | --- | --- | --- | --- | --- |
|  | Number of  Nucleotides (bp) | Number of  Nucleotides (bp) | Number of  Nucleotides (bp) | Number of  Nucleotides (bp) | Number of  Nucleotides (bp) | Number of  Nucleotides (bp) | Number of  Nucleotides (bp) | Number of  Nucleotides (bp) | Number of  Nucleotides (bp) | Number of  Nucleotides (bp) |
| 5´UTR | x | x | x | x | x | x | x | 200 | 50 | 45 |
| Exon 1 | x | x | x | x | x | x | x | 240 | 240 | 252 |
| Intron 1 | x | x | x | x | x | x | x | 114 | 127 | 122 |
| Exon 2 | x | x | x | x | x | x | x | 90 | 90 | 90 |
| Intron 2 | **1314** | x | x | x | x | x | x | 1554 | 1230 | 1666 |
| Exon 3 | **245** | x | x | x | x | x | x | 245 | 242 | 242 |
| Intron 3 | **433** | x | x | x | x | x | x | 1899 | 1536 | 300 |
| Exon 4 | **64** | x | x | x | x | x | x | 64 | 64 | 64 |
| Intron 4 | **286** | x | x | x | x | x | x | 174 | 177 | 188 |
| Exon 5 | **699** | x | x | x | x | x | x | 699 | 699 | 699 |
| Intron 5 | **88** | x | x | x | x | x | x | 94 | 162 | 89 |
| Exon 6 | **114** | 114 | x | x | x | 114 | 114 | 114 | 114 | 114 |
| Intron 6 | 111 | 111 | x | x | x | 111 | 92 | 110 | 109 | 103 |
| Exon 7 | 132 | 132 | x | *32* | *31* | 132 | 132 | 132 | 132 | 132 |
| Intron 7 | 143 | 154 | x | 143 | 144 | 140 | 139 | 175 | 86 | 133 |
| Exon 8 | 177 | 177 | x | 177 | 177 | 177 | 177 | 177 | 177 | 177 |
| Intron 8 | 478 | 328 | x | 478 | 318 | 329 | 343 | 314 | 1801 | 939 |
| Exon 9 | 54 | 54 | x | 54 | 54 | 54 | 54 | 54 | 54 | 54 |
| Intron 9 | 86 | 86 | x | 86 | 86 | 86 | 86 | 91 | 98 | 93 |
| Exon 10 | 63 | 63 | *30* | 63 | 63 | 63 | 63 | 63 | 63 | 63 |
| Intron 10 | 80 | 78 | 80 | 80 | 81 | 127 | 79 | 103 | 97 | 104 |
| Exon 11 | 936 | 936 | 936 | 936 | 936 | 936 | 936 | 912 | 903 | 903 |
| Intron 11 | 114 | 109 | 110 | 114 | 111 | 109 | 95 | 100 | 100 | 106 |
| Exon 12 | 128 | 128 | *28* | 128 | 128 | 128 | 128 | 128 | 128 | 128 |
| Intron 12 | **920** | 792 | x | 920 | 610 | 616 | 941 | 1144 | 2936 | 929 |
| Exon 13 | **277** | 277 | x | 277 | 277 | 277 | 277 | 283 | 277 | 277 |
| 3´ end | **422** | 344 | x | 168 | 185 | 163 | 103 | 3565 | 764 | 480 |

Note: Regions that could not be studied are shown with cross sign. For A genome number of nucleotides shown in bold represents sequence information obtained from flowsorted chromosome arm 3A sequences. Numbers with red background represents intronic sequences having gaps. Numbers in italic font represent partial exonic sequences.
