# Supplementary material for: Delineating the structural, functional and evolutionary relationships of sucrose phosphate synthase gene family II in wheat and related grasses
Source: BMC Plant Biol. 2010 Jun 30;10:134. doi: 10.1186/1471-2229-10-134 (PMC3017794; doi:10.1186/1471-2229-10-134)
Supplement: Additional file 2 — Sequence alignment of SPSII gene studied in all 10 genomes using MegAlign (ClustalW, slow/accurate). Boxes represented residues different from the consensus. TA(AA): Triticum aestivum A genome, TA(BB): Triticum aestivum B genome, TA(DD): Triticum aestivum D genome, TU: Triticum urartu, TS: Triticum speltoides, AT: Aegilops tauschii, HV: Hordeum vulgare, OS: Oryza sativa, SB: Sorghum bicolor, BD: Brachypodium distachyon. [file 1471-2229-10-134-S2.DOC]

Additional file 2

Sequence alignment of SPSII gene studied in all 10 genomes using MegAlign (ClustalW, slow/accurate). Boxes represented residues different from the consensus. TA(AA): Triticum aestivum A genome, TA(BB): Triticum aestivum B genome, TA(DD): Triticum aestivum D genome, TU: Triticum urartu, TS: Triticum speltoides, AT: Aegilops tauschii, HV: Hordeum vulgare, OS: Oryza sativa, SB: Sorghum bicolor, BD: Brachypodium distachyon.
