# Supplementary material for: Delineating the structural, functional and evolutionary relationships of sucrose phosphate synthase gene family II in wheat and related grasses
Source: BMC Plant Biol. 2010 Jun 30;10:134. doi: 10.1186/1471-2229-10-134 (PMC3017794; doi:10.1186/1471-2229-10-134)
Supplement: Additional file 3 — Sequence alignment of SPSII gene region containing six exons and six introns (intron 7 to exon 13) studied in nine genomes (without Triticum aestivum D genome). TA(AA): Triticum aestivum-A genome, TA(BB): Triticum aestivum-B genome, AT: Aegilops tauschii, HV: Hordeum vulgare, TU: Triticum urartu, TS: Triticum speltoides, BD: Brachypodium distachyon, SB: Sorghum bicolor, OS: Oryza sativa. Sequences from 5' UTR and 3' end are not shown due to unavailbilty of all sequeneces in some cases. Positions with SNP represented by grey boxes. Sequence in red font represented homoeologue-specific primers with 3' end SNP. Sequence in red font with underline represented overlapping forward and reverse homoeologue-specific primers with 3' end SNP. Unspliced intron in TA (BB) sequence is shown by bold italic font. TA(AA): Triticum aestivum A genome, TA(BB): Triticum aestivum B genome, TU: Triticum urartu, TS: Triticum speltoides, AT: Aegilops tauschii, HV: Hordeum vulgare, OS: Oryza sativa, SB: Sorghum bicolor, BD: Brachypodium distachyon. [file 1471-2229-10-134-S3.DOC]

Alignment Report of 'SPSII gene' - ClustalW (Slow/Accurate, IUB)

Majority --------------------------------------------------------------------------------

---------+---------+---------+---------+---------+---------+---------+---------+

10 20 30 40 50 60 70 80

---------+---------+---------+---------+---------+---------+---------+---------+

TA(AA) -------------------------------------------------------------------------------- 0

TA(BB) -------------------------------------------------------------------------------- 0

AT -------------------------------------------------------------------------------- 0

HV -------------------------------------------------------------------------------- 0

TU -------------------------------------------------------------------------------- 0

TS -------------------------------------------------------------------------------- 0

BD -------------------------------------------------------------------------------- 0

SB GTATGTATACAATTCTTTGTTGTTTGGCTTATTTTGTGCACATTACTGATGTATGTGCTCAGTAATGGTAATTGTTTTTC 80

OS -------------------------------------------------------------------------------- 0

Majority --------------------------------------------------------------------------------

---------+---------+---------+---------+---------+---------+---------+---------+

90 100 110 120 130 140 150 160

---------+---------+---------+---------+---------+---------+---------+---------+

TA(AA) -------------------------------------------------------------------------------- 0

TA(BB) -------------------------------------------------------------------------------- 0

AT -------------------------------------------------------------------------------- 0

HV -------------------------------------------------------------------------------- 0

TU -------------------------------------------------------------------------------- 0

TS -------------------------------------------------------------------------------- 0

BD -------------------------------------------------------------------------------- 0

SB CTGCAGACACTGATCATGGGCAATCGTGATGTCATTGATGAAATGTCAAGCACAAATGCAGCTGTTTTGACTTCAGTACT 160

OS -------------------------------------------------------------------------------- 0

Majority --------------------------------------------------------------------------------

---------+---------+---------+---------+---------+---------+---------+---------+

170 180 190 200 210 220 230 240

---------+---------+---------+---------+---------+---------+---------+---------+

TA(AA) -------------------------------------------------------------------------------- 0

TA(BB) -------------------------------------------------------------------------------- 0

AT -------------------------------------------------------------------------------- 0

HV -------------------------------------------------------------------------------- 0

TU -------------------------------------------------------------------------------- 0

TS -------------------------------------------------------------------------------- 0

BD -------------------------------------------------------------------------------- 0

SB CAAGTTAATTGATAAATACGATCTATATGGACAAGTGGCATACCCCAAGCACCACAAGCAATTTGAAGTTCCTGATATTT 240

OS -------------------------------------------------------------------------------- 0

Majority --------------------------------------------------------------------------------

---------+---------+---------+---------+---------+---------+---------+---------+

250 260 270 280 290 300 310 320

---------+---------+---------+---------+---------+---------+---------+---------+

TA(AA) -------------------------------------------------------------------------------- 0

TA(BB) -------------------------------------------------------------------------------- 0

AT -------------------------------------------------------------------------------- 0

HV -------------------------------------------------------------------------------- 0

TU -------------------------------------------------------------------------------- 0

TS -------------------------------------------------------------------------------- 0

BD -------------------------------------------------------------------------------- 0

SB ATCGTTTAGCTGCAAGAACAAAAGTAACTGAGCCTTCTATTTTTTAATGCATTAAGATGTTTCAATGTGATGGGAGTGCT 320

OS -------------------------------------------------------------------------------- 0

Majority --------------------------------------------------------------------------------

---------+---------+---------+---------+---------+---------+---------+---------+

330 340 350 360 370 380 390 400

---------+---------+---------+---------+---------+---------+---------+---------+

TA(AA) -------------------------------------------------------------------------------- 0

TA(BB) -------------------------------------------------------------------------------- 0

AT -------------------------------------------------------------------------------- 0

HV -------------------------------------------------------------------------------- 0

TU -------------------------------------------------------------------------------- 0

TS -------------------------------------------------------------------------------- 0

BD -------------------------------------------------------------------------------- 0

SB AGTGGATTCCATCTTATCTTCTTTGAGATTAGTTACCCTCTATCTCAAATTGCAAGTTGTTTTAGTTTTGTCCAAAGTCA 400

OS -------------------------------------------------------------------------------- 0

Majority --------------------------------------------------------------------------------

---------+---------+---------+---------+---------+---------+---------+---------+

410 420 430 440 450 460 470 480

---------+---------+---------+---------+---------+---------+---------+---------+

TA(AA) -------------------------------------------------------------------------------- 0

TA(BB) -------------------------------------------------------------------------------- 0

AT -------------------------------------------------------------------------------- 0

HV -------------------------------------------------------------------------------- 0

TU -------------------------------------------------------------------------------- 0

TS -------------------------------------------------------------------------------- 0

BD -------------------------------------------------------------------------------- 0

SB AACTTGTTGAACTTTGACCAAATTTATAGAATATTATATTTTTTTCTCGAACAACGCAGGAGAGCTGCGTGTCATTTCAT 480

OS -------------------------------------------------------------------------------- 0

Majority --------------------------------------------------------------------------------

---------+---------+---------+---------+---------+---------+---------+---------+

490 500 510 520 530 540 550 560

---------+---------+---------+---------+---------+---------+---------+---------+

TA(AA) -------------------------------------------------------------------------------- 0

TA(BB) -------------------------------------------------------------------------------- 0

AT -------------------------------------------------------------------------------- 0

HV -------------------------------------------------------------------------------- 0

TU -------------------------------------------------------------------------------- 0

TS -------------------------------------------------------------------------------- 0

BD -------------------------------------------------------------------------------- 0

SB GAAGAAGAAGAAATAGTGCACAAAGGAGGGGGATTACTCCCTCCAAAAAAACACCCCATACACAATGGGAAAGAAAAATA 560

OS -------------------------------------------------------------------------------- 0

Majority --------------------------------------------------------------------------------

---------+---------+---------+---------+---------+---------+---------+---------+

570 580 590 600 610 620 630 640

---------+---------+---------+---------+---------+---------+---------+---------+

TA(AA) -------------------------------------------------------------------------------- 0

TA(BB) -------------------------------------------------------------------------------- 0

AT -------------------------------------------------------------------------------- 0

HV -------------------------------------------------------------------------------- 0

TU -------------------------------------------------------------------------------- 0

TS -------------------------------------------------------------------------------- 0

BD -------------------------------------------------------------------------------- 0

SB CACCACCACCCTGAGCACAGAAACAGACTCACTCCATTAGTGATCTAGAACGAGTGACCTAGCAAGGTGCTCATGCAACT 640

OS -------------------------------------------------------------------------------- 0

Majority --------------------------------------------------------------------------------

---------+---------+---------+---------+---------+---------+---------+---------+

650 660 670 680 690 700 710 720

---------+---------+---------+---------+---------+---------+---------+---------+

TA(AA) -------------------------------------------------------------------------------- 0

TA(BB) -------------------------------------------------------------------------------- 0

AT -------------------------------------------------------------------------------- 0

HV -------------------------------------------------------------------------------- 0

TU -------------------------------------------------------------------------------- 0

TS -------------------------------------------------------------------------------- 0

BD -------------------------------------------------------------------------------- 0

SB TGGAGGACCCTGCCAAGCACCACATGGTACTCTCATTGGCAACTGATTGCAGTAGCACTTCAATGTTAGGTCTAGCACCC 720

OS -------------------------------------------------------------------------------- 0

Majority --------------------------------------------------------------------------------

---------+---------+---------+---------+---------+---------+---------+---------+

730 740 750 760 770 780 790 800

---------+---------+---------+---------+---------+---------+---------+---------+

TA(AA) -------------------------------------------------------------------------------- 0

TA(BB) -------------------------------------------------------------------------------- 0

AT -------------------------------------------------------------------------------- 0

HV -------------------------------------------------------------------------------- 0

TU -------------------------------------------------------------------------------- 0

TS -------------------------------------------------------------------------------- 0

BD -------------------------------------------------------------------------------- 0

SB TCAAACACACAAGCATTCTGATGCTTCCATAATTCCCAAGCAACTAGGATGATTAGAGTATTGAGGCCCTTGCCCTTATT 800

OS -------------------------------------------------------------------------------- 0

Majority --------------------------------------------------------------------------------

---------+---------+---------+---------+---------+---------+---------+---------+

810 820 830 840 850 860 870 880

---------+---------+---------+---------+---------+---------+---------+---------+

TA(AA) -------------------------------------------------------------------------------- 0

TA(BB) -------------------------------------------------------------------------------- 0

AT -------------------------------------------------------------------------------- 0

HV -------------------------------------------------------------------------------- 0

TU -------------------------------------------------------------------------------- 0

TS -------------------------------------------------------------------------------- 0

BD -------------------------------------------------------------------------------- 0

SB TTTTTGAACACTTTTAATAGTGCAACACCACCAGCAGGAGAAACTATCACTCATCTCTTGACGAGCAAGAGTCAGCAGGT 880

OS -------------------------------------------------------------------------------- 0

Majority --------------------------------------------------------------------------------

---------+---------+---------+---------+---------+---------+---------+---------+

890 900 910 920 930 940 950 960

---------+---------+---------+---------+---------+---------+---------+---------+

TA(AA) -------------------------------------------------------------------------------- 0

TA(BB) -------------------------------------------------------------------------------- 0

AT -------------------------------------------------------------------------------- 0

HV -------------------------------------------------------------------------------- 0

TU -------------------------------------------------------------------------------- 0

TS -------------------------------------------------------------------------------- 0

BD -ACACTGATTATGGGTAACCGTGATGTTATTGATGAAATGTCAAGCACAAATGGAGCTGTTTTGACATCAGTACTCAAGT 79

SB CGAGCCTCTGGAAAATTAAAGCCCAAGCTTGCCTAGCGAACACACAAGAAATGAGGATGTGCTGGATATTTTCCTCCGCC 960

OS -------------------------------------------------------------------------------- 0

Majority --------------------------------------------------------------------------------

---------+---------+---------+---------+---------+---------+---------+---------+

970 980 990 1000 1010 1020 1030 1040

---------+---------+---------+---------+---------+---------+---------+---------+

TA(AA) -------------------------------------------------------------------------------- 0

TA(BB) -------------------------------------------------------------------------------- 0

AT -------------------------------------------------------------------------------- 0

HV -------------------------------------------------------------------------------- 0

TU -------------------------------------------------------------------------------- 0

TS -------------------------------------------------------------------------------- 0

BD TGATTGACAAATACGATCTATATGGTCAAGTGGCATACCCCAAGCACCATAAGCAATCTGATGTCCCAGATATTTACCGT 159

SB TGGTCACAAAGCGGACGCCGCTGGATGAGGAAGCCCGCGCTTAGGAAGATGATTGGCTGTCTACCACCGATTTTTAATGG 1040

OS -------------------------------------------------------------------------------- 0

Majority --------------------------------------------------------------------------------

---------+---------+---------+---------+---------+---------+---------+---------+

1050 1060 1070 1080 1090 1100 1110 1120

---------+---------+---------+---------+---------+---------+---------+---------+

TA(AA) -------------------------------------------------------------------------------- 0

TA(BB) -------------------------------------------------------------------------------- 0

AT -------------------------------------------------------------------------------- 0

HV -------------------------------------------------------------------------------- 0

TU -------------------------------------------------------------------------------- 0

TS -------------------------------------------------------------------------------- 0

BD TTGGCAGCAAGAACCAAGGTAACCGAGTCTTATATCTTCATATTTTGAAATATGTGAAAATAGAGTTCTGCAATAAGCAT 239

SB CCAACCAAATGAAGAATTTAAATCTCAAAGGTGCCCAGCTTTTCTAGATGTGCTTCCAAGGTGCAAACAGCAAACCTAAT 1120

OS -------------------------------------------------------------------------------- 0

Majority -----------------------------------GTATGTGCCAGTCCTGTGTAGTTTCTCTTGTTGCCTTAATTA---

---------+---------+---------+---------+---------+---------+---------+---------+

1130 1140 1150 1160 1170 1180 1190 1200

---------+---------+---------+---------+---------+---------+---------+---------+

TA(AA) -----------------------------------GTATGTGCCAGTACTGTGTAGTTTCTCTTGTTGCCTTAATTA--- 42

TA(BB) -----------------------------------GTATGTGCCAGTCCTGTGTAGTTTCTCTTGTTGCCTTAACTAATA 45

AT -----------------------------------GTATGTGCCAGTCCTGTGTAGTTTCTCTTGTTGCCTCAATTA--- 42

HV -----------------------------------GTATGTGCCAGTCCTGTGTAGTTTCTCTTGTTGCCTTAATT---- 41

TU -----------------------------------GTATGTGCCAGTACTGTGTAGTTTCTCTTGTTGCCTTAATTA--- 42

TS ----ACCAGGAACTGAGAAATTTAGCAACAACCTTGTATGTGCCAGTCCTGTGTAGTTTCTCTTGTTGCCTTAACTAA-- 74

BD GCTACCGAATTCTCTGTGGTACCATGATTAATCGTTAATTGGATGTTATAGATTAAGTGGTAATTAAGTTTTAACCTGTC 319

SB ACTTCCCAGGAAGAAAGCTTACATAAGCCGACTTGCTGCTGTAAAGCCCAGAATTTGTTAGCTT-CCACCTATGTTGATC 1199

OS ---------------------------------GTGCGTGAGCTTGTCTAATGTGGTTTGCCTAGTTGCCCTCATTCTTA 47

Majority TAT-----ATTTTTTX-------TGCTA--TACGGCGGTTTAGTTATCATTAATAATCTTC--------TGC---CACCA

---------+---------+---------+---------+---------+---------+---------+---------+

1210 1220 1230 1240 1250 1260 1270 1280

---------+---------+---------+---------+---------+---------+---------+---------+

TA(AA) TAT-----ATTTTTT--------TGCTA--TACGGCGGTTTAGTTATCATTAATAATCTTC--------TGC---CATCA 96

TA(BB) TAT-----ATTTTTTATATTTTCTGCTA--TCCGGCGGTTTAGTTATCATTAATAATCTTC--------TGC---CACCA 107

AT --------ATTTTTT--------TGCTA--TCCAGCCGTTTAGTTATCATTAATAATCTTC--------TTC---CACCA 93

HV ---------TTCTTTA-------TGCTA--TCCGGCAGTTCAGTTATCNTTAATANTCTTC--------TGC---CACCA 92

TU TAT-----TTTTTTT--------TGCTA--TACGGCGGTTTAGTTATCATTAATAATCTTC--------TGC---CATCA 96

TS TAA-----TTATTTT--------TGCTA--TCCGGCGGTTTAGTTATCATTAATAATCTTC--------TGC---CACCA 128

BD TGT-----ATTTATTA--GTTTTTGGAA--AAGGAAGAGTTGTTTTTCCTTGGGCCTCTGCGTATGAAATGC---ACACA 387

SB GGGGACCTCTGGTTGTAAAACCAAACCATCGACCAGATCCTATATTCTCTTAGCACTTTCCCTGTAATATCCGCAACCCA 1279

OS TTTTTCTCATTATCTAGTGGTCTAGTTAG-TAATGAAGCTTCTATGTCCTTTCTAGGTGATCATTCTTTTTG---TAGTA 123

Majority ACTATCACTGCCTGTTCCC-AGTC-----TTGATAACTATTCTTTATCGGCAGACATTGATCATGGGTAACCGTGATGTT

---------+---------+---------+---------+---------+---------+---------+---------+

1290 1300 1310 1320 1330 1340 1350 1360

---------+---------+---------+---------+---------+---------+---------+---------+

TA(AA) ACTATCACTGCCTGTTCCC-AGTC-----TTTATAACTATTCTTTATCGGCAGACGTTGATCATGGGTAACCGTGATGTT 170

TA(BB) ACTATCACTGCCTGTTCCC-GGTC-----TTGATAACTATTCTTTATCGGCAGACATTGATCATGGGTAACCGTGATGTT 181

AT ACTATCACTGCCTGTTCCC-AGTC-----TTGATAACTATTCTTTATCGGCAGACATTGATCATGGGTAACCGTGATGTT 167

HV ACTATCACTGCCTGTTCTC-AGTC-----TTGATAACTATTCTTTATCGGCAGACATTGATCATGGGTAACCGTGATGTT 166

TU ACTATCACTGCCTGTTCCC-AGTC-----TTTATAACTATTCTTTATCGGCAGACGTTGATCATGGGTAACCGTGATGTT 170

TS ACGATCACTGCCTGTTCCC-AGTC-----TTGATAACTATTCTTTATCGGCAGACATTGATCATGGGTAACCGTGAGGTT 202

BD GTTTACGCAGTCAGGAGCC-AATCAGCATCACATAAGTGAAATGAGTACGTAAAAGCTGACAGTGT-TATCTCAGGCATT 465

SB GCCCCTATTGTCTAAGGCTTGAGCCACAGTACGTTGTTTCACTGCTTGTTTGGAGATCAGTT-TACACAAATTAGGTTCT 1358

OS TTTTTTATACTCTCTAGAT-GATGCTTGTTCTCTTCATTTATTTTTTCTGCAGACACTTATCATGGGTAACCGTGATGTT 202

Majority AT-TGATGAAATGTCAAGCACAAATGGA-GCTGTTTTGACATCAGTACTC-------AAGTTAATTGACAAGTATGATCT

---------+---------+---------+---------+---------+---------+---------+---------+

1370 1380 1390 1400 1410 1420 1430 1440

---------+---------+---------+---------+---------+---------+---------+---------+

TA(AA) AT-TGATGAAATGTCAAGCACAAATGGA-GCTGTTTTGACATCAGTACTC-------AAGTTAATTGACAAGTATGATCT 241

TA(BB) AT-TGATGAAATGTCAAGCACAAATGGA-GCTGTTTTGACATCAGTACTC-------AAGTTAATTGACAAGTATGATCT 252

AT AT-TGATGAAATGTCAAGCACAAATGGA-GCTGTTTTGACATCAGTACTC-------AAGTTAATTGACAAGTATGATCT 238

HV AT-TGATGAAATGTCAAGCACAAATGGA-GCTGTTTTGACATCAGTACTC-------AAGTTAATTGACAAGTATGATCT 237

TU AT-TGATGAAATGTCAAGCACAAATGGA-GCTGTTTTGACATCAGTACTC-------AAGTTAATTGACAAGTATGATCT 241

TS AT-TGATGAAATGTCAAGCACAAATGGA-GCTGTTTTGACATCAGTACTC-------AAGTTAATTGACAAGTATGATCT 273

BD ACATGGTCTGGAGAAAGTACCCGGTGGATACCATCTTACAGTAAAAACTCCGAATGGAAACATGTTTAAAGTTTTCACAA 545

SB AGCTCAAATAATGTCTTGCCT---TGCA-ACCATCTATTAGTCGAGAATTTAGTTGAAAACACGTTGCCAACTAGAGTTT 1434

OS AT-TGATGAAATGTCAAGCACAAATTCA-GCTGTTTTGACATCAATACTC-------AAGTTAATTGACAAGTATGATCT 273

Majority ATATGGGCAAGTGGCATACCCCA--AGCACCATAAGCAATCTGAAGTTCCAGAT--ATTTATCGTTTAGCGGCAAGAACA

---------+---------+---------+---------+---------+---------+---------+---------+

1450 1460 1470 1480 1490 1500 1510 1520

---------+---------+---------+---------+---------+---------+---------+---------+

TA(AA) ATATGGGCAAGTGGCATACCCCA--AGCACCATAAGCAATCTGAAGTTCCAGAT--ATTTATCGTCTAGCGGCAAGAACA 317

TA(BB) ATATGGGCAAGTGGCATACCCCA--AGCACCATAAGCAATCTGAAGTTCCAGAT--ATTTATCGTTTAGCGGCAAGAACA 328

AT ATATGGGCAAGTGGCATACCCCA--AGCACCATAAGCAATCTGAAGTTCCAGAT--ATTTATCGTTTAGCGGCAAGAACA 314

HV CTATGGGCAAGTGGCATACCCCA--AGCACCATAAGCAATCTGAAGTTCCAGAT--ATTTATCGTTTAGCGGCAAGAACA 313

TU ATATGGGCAAGTGGCATACCCCA--AGCACCATAAGCAATCTGAAGTTCCAGAT--ATTTATCGTCTAGCGGCAAGAACA 317

TS ATATGGGCAAGTGGCATACCCCA--AGCACCATAAGCAATCTGAAGTTCCAGAT--ATTTATCGTTTAGCGGCAAGAACA 349

BD ATTAAGAAAAAAAATGTATGTTAGGAGAGTGGTTTTAGATGCAAAATTTCAAGT--TCAAACTCATTCATTTCAGGGGTA 623

SB CTGCAGCCACAATGAAGAGGGGCCTGGCATTTTGTGTCACTTGGATTGGCAGACCAGTCCATGAACATGATGCACCTGTT 1514

OS ATATGGCCAAGTGGCATACCCCA--AGCACCATAAGCAATCTGAAGTTCCAGAT--ATTTATCGTTTAGCGGCAAGAACA 349

Majority AA-GGTAACCAAGTGT---ATCTTTATATTTTGAAATGTTT---CG--GTGGCCTTCTATAATAAGCXTGCTAGTGGATT

---------+---------+---------+---------+---------+---------+---------+---------+

1530 1540 1550 1560 1570 1580 1590 1600

---------+---------+---------+---------+---------+---------+---------+---------+

TA(AA) A---GTAACCAAGTGT---ATCTTTATATTTTGAAATGTTT---CG--GTGGCCTTATATAATAAGTGTGCTAGTGGATT 386

TA(BB) AA-GGTAACCAAGTGT---ATCTTTATATTTTGAAATGTTT---CG--GTGGCCTTCTATAATAAGCATGCTAGTGGATT 399

AT AA-GGTAACCAAGTGT---ATCATTATATTTTGAAATATTT---CG--GTGGCCTTCTATAATAAGCATGCTAGTTGATT 385

HV AA-GGTAACCAAGTGT---ATCTTTATATTTTGAAATGTTT---TG--GCGGCCTTCTATAATAAGCGTGCTAGTGGGTT 384

TU AA-GGTAACCAAGTGT---ATCTTTATATTTTGAAATGTTT---CG--GTGGCCTTCTATAATAAGTGTGCTAGTGGATT 388

TS AA-GGTAAGCAAGTGT---ATCTTTGTATTTTGAAATGTT----CG--GTGGCCTTCTATAATAAGCGTGCTAGTGGATT 419

BD TGTAAAAGACAAGTTC---AGCAATAAATAGTAGCATCTCTGTTTG--GTACTATTCATGGTTAAATTTGTTTTTTCATA 698

SB TTTTGTGCCCAAAGGCAGTGTTTCTAGGTTATCAATTCCAAGACCGCCATAGTATAGGGCGCTGGACAAGCTCCTTAGAC 1594

OS AA-GGTAGTCAAATATTATATCTTCATATGTTGAAATGTTT---CA--TATGGTTTCTGCAATAAGTATTCTAGTGGACT 423

Majority CTCT------GACATC--GTATCATAA-TTAAGTTATGATTGGATGCAATGGGCCTACTGGTAXTCA--CATTTTXAACT

---------+---------+---------+---------+---------+---------+---------+---------+

1610 1620 1630 1640 1650 1660 1670 1680

---------+---------+---------+---------+---------+---------+---------+---------+

TA(AA) CTCT------GACATC--GTATCATAA-TTAAGTTATGATTGGATGCAATGGGCCTACTGGTTCTCA--CATTTT-AACT 454

TA(BB) CTCT------GACATC--GTATCATAA-TTAAGTTATGACTGGATGCAATGGGTCTACTGATAATCA--CATTTT-AACT 467

AT CTCT------GACATT--GTATCATAA-TTAAGTTATGAATGGATGCAATGGGCCTACTGGTAGTCA--CATTTT-AACT 453

HV CTCT------GACAT---GTATCATAA-TTAAGTTATGATTGGATGCAATGGGCCTACTGGTAGTCA--CATTTT-AACT 451

TU CTCT------GACATC--GTATCATAA-TTAAGTTATGATTGGATGCAATGGGCCTACTGGTTCTCA--CATTTTTAACT 457

TS CTCT------GACTTC--GTATAATAA-TTAAGTTATGATTGGATGCAATGGGCCTACTGGTAGTCA--CATTTTTAACT 488

BD CTTTCCAAATGAATTTGAGTTTGAGAACTTGAAATTTTATATGTTATTATGGAACTCCTCTCCCCCAA-CATATTGTATT 777

SB ACGAGACATGGAGAAGATCTCTCAAAGATGAAGCTCGACTTCAAGCAACTAGGATTTCTGAAAATAAGCGATCTGTTTTT 1674

OS CTT-------GACATGTGGTTCTTGAGATT-AGTTGTATGGTGAT-CCAGATAAGTGTTGACAATAG--GATTTATGATG 492

Majority ATCTGTCAGTAT--------------------------------------T-TAGTTA----------XCXGATGCATAT

---------+---------+---------+---------+---------+---------+---------+---------+

1690 1700 1710 1720 1730 1740 1750 1760

---------+---------+---------+---------+---------+---------+---------+---------+

TA(AA) ATCTGTCAGTATTACTCCCTCCGTTCCTAAATATAAGTCTTTCTAAGAGATTTCATTATGGACTACGTACGGATGTATAT 534

TA(BB) ATCTGTTA------------------------------------------T-TAGTTA----------GCTGATGCATAT 494

AT ATCTGTCAGTAT----------------------------------------TAGTTA----------GCTGATGCATAT 483

HV ATCTGTCAGTAT----------------------------------------TAGTTA----------TCGGATGCAAAT 481

TU ATCTGTCAGTATTACTCCCTCCGTTCCTAAATATAAGTCTTTCTA-GAGATTTAATTATGGACTACGTACGGATGTATAT 536

TS GTCTGTCAGTAT----------------------------------------TAGTTG----------GCTGATGCATAT 518

BD TTTTAAAAACTTAACATGTTTCTTGTGTGGGT-------CTTCACCAGTTTCCACCTGTAAGGTGGTTTCCATTGGATAT 850

SB CTCTCCTGGGTTGATTCTTGTGTTTAGTGGTTCTTTGTTTTTCTTCTTGTTCTAGCCTTGATA-GGGCTTAGCTGAAGTG 1753

OS AGCCATATGC--------------------------------------------ATTG----------ATAGTTACATAA 518

Majority C-----------------------------TTGTGTTCTA-----A----ATTTTAGX-----------GGCT-------

---------+---------+---------+---------+---------+---------+---------+---------+

1770 1780 1790 1800 1810 1820 1830 1840

---------+---------+---------+---------+---------+---------+---------+---------+

TA(AA) GAACATAGTT-TAGAGTGTAGATTGACTCATTTTGCTCCGTATGTAATCTATATTAGAATCTCTAGAAAGACTTATATTT 613

TA(BB) C-----------------------------TTGTGTTCTA-----A-----TTTTAGT-----------GGCT------- 517

AT C-----------------------------TTGTGTTCTA----------ATTTTAGT-----------GGCT------- 506

HV C-----------------------------TTGTGTTCTA----------ATTTTAAT-----------GGCT------- 504

TU GAACATAGTT-TAGAGTGTAGATTCACTCATTTTGCTCCGTATGTAGTCTATATTAGAATCTCTAGAAAGACTTATATTT 615

TS C-----------------------------TTGTGTTCTA----------ATTTTAAG------------GCT------- 540

BD TTTCTCCATAGTCTAATGTGCGATCGCTGGCCATGTTGGGACATAAATCCCCTGTCTACTGACTCCAGCTTGTCGCCACC 930

SB TTTTCCGTTTGTTTGGCTCAGTGTTTATTTTATTGTTTTAACAAAATCTAACAGTGGGCGCTTCCCTGCTGTTTTCCTGG 1833

OS TT---------------------------GTCATTTTGGA--------------TAT----------------------- 534

Majority -----------------TTXGCT--------T-TTG-XTTX-TXATTTT------------TXCTT---------TCTTT

---------+---------+---------+---------+---------+---------+---------+---------+

1850 1860 1870 1880 1890 1900 1910 1920

---------+---------+---------+---------+---------+---------+---------+---------+

TA(AA) AGGAACGGAGGGAGTAGTTAGCTGATGCATATCTTGTGTTC-TAATTTTAATGGCTTTGGCAATTTGCCTA--G-TCTTT 689

TA(BB) -----------------TTAGCT--------TTTTGACTTG-TTGTTTT------------TGCTT---------CCTTT 550

AT -----------------TTGGTT--------TTTTGACTTG-TTATTTT------------TGCTT---------CCTTT 539

HV -----------------TTAGCT----------------------TTTT------------GACTTGT-------CTTTC 526

TU AGGAACGGAGGGAGTAATTAGCTGATGCATATCTTGTGTTC-TAATTTTAATGGCTTTGGCAATTTGCCTA--G-TCTTT 691

TS -----------------TTGGCT----------------------TTTT------------GACTT-------G-TCTTT 561

BD AACCACCAGAGACCCCCATGCCTCCCATCGTTGTAGCGAAGACTATGTTGGCATCTTTACTTGTTTTTCTCCTC-TCTTT 1009

SB TTAAAAAAAAATGGAGTATTATTTTATCAAAAATCACTATCAGGCCGTGCAGGTGATAGTATGTCTAACTTGGCAGTGTT 1913

OS -----------------TTGCTT----------------------------------------CCT---------TTTAC 548

Majority TTTGCTCAG--TCTGACT------TGCAGCAACAAACTGACCGTGCTGCTTCTTTGTGC--CTTACTCACAT--TTT---

---------+---------+---------+---------+---------+---------+---------+---------+

1930 1940 1950 1960 1970 1980 1990 2000

---------+---------+---------+---------+---------+---------+---------+---------+

TA(AA) TTTGCTCAC--TCTGACT------TGCAGCAACAAACTGACCGTGCTGCTTCTTTGTGC--CCTACTCACAT--TTT--- 754

TA(BB) TTTGCTCAG--TCTGACT------TGCAGCAACAAACTGACCGTGCTGCCTCTTTGTGC--CTTACTCACATGTTTT--- 617

AT TTTGCTCAG--TCTGACT------TGCAGCAACAAACTGACCGTGCTGCTTCTTTGTGG--CTTACTCACAT--TTT--- 604

HV TTTGCTCAG--TCTGACT------TGCAGAAACAAACTGACCGTGCTGCTT-TTTGTGC--CTTACTCACAT--TTC--- 590

TU TTTGCTCAC--TCTGACT------TGCAGCAACAAACTGACCGTGCTGCTTCTTTGTGC--CCTACTCACAT--TTT--- 756

TS TTTGCTCAG--TCTGACT------TGCAGCAAGAAACTGACCGTGCTGCTTCTTTGTGC--TTTACTCACAT--TTT--- 626

BD TTTGCTTAAGTTCTGACT------TTCAGCAACAAACGGAC-ATGCTTTTTGTTTGTGC--CTTACTCACAT--TTC--- 1075

SB GATGACTAGTTTTTGATAATCTTATGTGTTATCTGATGAACTTTGGTGTTTGCTAGTATAATTCAATTTTATTTTTTGTA 1993

OS CTATCCCAG--TTTTACT------TTCAGCAAGGAACTGATTATGCTGCTTCTATGCTT--CTTTATGCCAGCCTCA--- 615

Majority --TATACATAAATATAT---------------------------GCTTAT-TTTAGTTTCTGTTGTTTCCA--GGGGGTG

---------+---------+---------+---------+---------+---------+---------+---------+

2010 2020 2030 2040 2050 2060 2070 2080

---------+---------+---------+---------+---------+---------+---------+---------+

TA(AA) --TATACATAAATATAT---------------------------GCTTAT-TTTAGTTTCTGTTGTTTCCAGAGGGGGTG 804

TA(BB) --TATACATAAATATAT---------------------------GCTGAT-TTTAGTTTCTGTTGTTTCCA--GGGGGTG 665

AT --TATACATAAATATAT---------------------------GCTTAT-TTCAGTTTCTGTTGTTTCCA--GGGGGTG 652

HV --TATACATAAATATATTACTCACATTTCTATACATAAATATATGCTTAT-TTTAGTTTCTGTTGTTTCCA--GGGGGTG 665

TU --TATACATAAATATAT---------------------------GCTTAT-TTTAGTTTCTGTTGTTTCCA--GGGGGTG 804

TS --TATACATAAATATATA-------------------------TGCTTAT-TTTAGTTTCTGTTGTTTCCA--GGGGGTG 676

BD --CATAC-TAAAAACAT---------------------------GCTCAT-CTGAGTTCCTATTATTTTCA--GGGGGTG 1122

SB ATTTTACTTTAAGAAATCTACTGATTGATTCTTGACTTTCACA-ACTAATGTTGAGTTTCAATTATTTTCA--GGGAGTT 2070

OS --ATCACATCTTCATGTT-----------------TGAACTAATTATTGT-TTGAATCT-TATCAATTTCA--GGGGGTG 672

Majority TTTATTAACTGTGCTTATATTGAACCATTTGGGCTCACCTTGATCGAGGTT----ACTTCTGCAACATTTCATTTTGTTG

---------+---------+---------+---------+---------+---------+---------+---------+

2090 2100 2110 2120 2130 2140 2150 2160

---------+---------+---------+---------+---------+---------+---------+---------+

TA(AA) TTTATTAACTGTGCTTATATTGAACCATTTGGGCTCACCTTGATCG--GTT----ACTTCTGCAACATTTCATTTTATTG 878

TA(BB) TTTATTAACTGTGCTTATATTGAACCATTTGGGCTCACCTTGATCG--GTT----ATTTCTGCAACATTTCATTTTGTTG 739

AT TTTATTAACTGTGCTTATATTGAACCATTTGGGCTCACCTTGATTGAGGTT----ACTTCTGCAACATTTCATTTTGTTG 728

HV TTTATTAACTGTGCTTATATTGAACCATTTGGGCTCACCTTGATCGAGGTT----ACTTCTGCAACATTTTATTTTGTTG 741

TU TTTATTAACTGTGCTTATATTGAACCATTTGGGCTCACCTTGATCGAGGTT----ACTTCTGCAACATTTCATTTTATTG 880

TS TTTATTAACTGTGCTTATATCGAACCATTTGGGCTCACCTTGATCGAGGTT----ACTTCTGCAACATTTCATTTTGTTG 752

BD TTTATTAACCCAGCTTATATTGAACCATTTGGACTCACCTTGATCGAGGTTCT-TACTTCTGCAACATTTCCTTTGGCTG 1201

SB TTTATCAATTGTGCATTCATTGAACCATTTGGACTCACCTTGATTGAGGTGGTTACTTTTTGCAATATTTCCTTTGCTTA 2150

OS TTTATCAATTGTGCTTTTATCGAGCCATTTGGACTAACCTTGATTGAGGTTATGTACTGCTGTGGGATTTCCTGTTGTTG 752

Majority TTA----CTTTATCTGTGGAT---TGATAATGTTTATGCAAGCTTTACACCTTGTTGCCTGCC-AGGCTGCTGCTTATGG

---------+---------+---------+---------+---------+---------+---------+---------+

2170 2180 2190 2200 2210 2220 2230 2240

---------+---------+---------+---------+---------+---------+---------+---------+

TA(AA) TTA----CTCTATCTGTGGAT---TGATAATGTTTATGCAAGCTTTACATCTTGTTTGCTGC--AGGCTGCTGCTTATGG 949

TA(BB) TTA----CTTTATCTGTGGAT---TGATAATGTTTATGCAAGCTTTACACCTTGTTGCCTGCC-AGGCTGCTGCTTATGG 811

AT TTA----CTTTATCTGTGGAT---TGATAATGTTTATGCAAGCTTTACACCTTGTTGCCTGCC-AGGCTGCTGCTTATGG 800

HV TTA----CTTTATCTGTGGAT---TGATGATGTTTATGCAAGCTTTACACCTTGTTGCATGCC-AGGCTGCTGCTTATGG 813

TU TTA----CTTTATCTGTGGAT---TGATAATGTTTATGCAAGCTTTACATCTTGTTGCCTGCC-AGGCTGCTGCTTATGG 952

TS TTA----CTTTATCTGTGGAT---TGATAATATTTATGCAAGCTTTACACCTTGTTGCCTGCC-AGGCCGCTGCTTATGG 824

BD TTAGTTACTTTATGTGTTGAT---TAACAATGTTTATGCAATCTTTGCACCTTGTTGCCTCTC-AGGCTGCTGCTTATGG 1277

SB CTTACTTATGCCCATGTTAATGTTTGCTAGTTGGTATTTGATCTTTACTTCTTTTTTCCTTTCTAGGCTGCTGCTTATGG 2230

OS CTCA--ACTTTATATGTGAAT---TAAT-ATGCTTATGCAATCTTAACATCTTGTTGGTTGGC-AGGCTGCTGCTTATGG 825

Majority TCTACCTATGGTTGCTACCCAAAATGGTGGGCCTGTCG------------------------------------------

---------+---------+---------+---------+---------+---------+---------+---------+

2250 2260 2270 2280 2290 2300 2310 2320

---------+---------+---------+---------+---------+---------+---------+---------+

TA(AA) TCTACCTATGGTTGCTACCCAAAATGGTGGGCCTGTCG------------------------------------------ 987

TA(BB) TCTACCTATGGTTGCTACCCAAAATGGTGGACCTGTCG------------------------------------------ 849

AT TCTACCTATGGTTGCTACCCAAAATGGTGGGCCTGTCGTTTTTTTTTTTTGTTATATGGTTGCTACCCAAAATGGTGGGC 880

HV TCTACCTATGGTTGCTACCCAAAATGGTGGGCCTGTTG------------------------------------------ 851

TU TCTACCTATGGTTGCTACCCAAAATGGTGGGCCTGTCG------------------------------------------ 990

TS TCTACCTATGGTTGCTACCCAAAATGGTGGGCCTGTCG------------------------------------------ 862

BD TCTACCGATGGTTGCTACACAAAATGGTGGACCTGTAG------------------------------------------ 1315

SB TCTACCCATGGTTGCCACCCGAAATGGTGGGCCTGTGG------------------------------------------ 2268

OS TCTACCAATGGTTGCTACTCGAAATGGTGGGCCCGTTG------------------------------------------ 863

Majority ------ATATACACCGGGTACTGTTCTGCTATCTTGGTTTAXGTTTTGCAGTGACTCACTTTTTGTTCTGGCTATTGATG

---------+---------+---------+---------+---------+---------+---------+---------+

2330 2340 2350 2360 2370 2380 2390 2400

---------+---------+---------+---------+---------+---------+---------+---------+

TA(AA) ------ATATACACCGGGTACTGTTCTGCTATCTTGGTTTACGTTTTGCAGTGACTCACTTTTTGTTCTGCCTATTGATG 1061

TA(BB) ------ATATACACCGGGTACTGTTCTGCCATCTTAGTTTACGTTTTGCAGCGACACACTTTTTGTTCTGGCTATTGATG 923

AT CTGTTGATATACACCGGGTACTGTTCTGCTATCTTGGTTTATGTTTCACAGTGACTCACTTTTTGTTCTGGCTATTGATG 960

HV ------ATATACACCGGGTACTGTTCTGCTATCTTGGTTTATGTCTCACAGTGACTCACTTTTT-TTCTGGCTATTGATG 924

TU ------ATATACACCGGGTACTGTTCTGCTATCTTGGTTTACGTTTTGCAGTGACTCACTTTTTGTTCTGCCTATTGATG 1064

TS ------ATATACACCGGGTACTGTTCTGCCATCTTGGTTTACGTTTTGCAGTGACGCACTTTTTGTTCTGGCTATTGATG 936

BD ------ACATACATCGGGTACTGCTGTGCTATCTTGGTTTATCTTTTGCACTGATTTTTTTTTC-CTTTGCTTATTGATG 1388

SB ------ACATACATCGGGTATTGATGCACATGCTTTCTTTGTTACTGTGGGCAAA---TTTCCTGCTTGAGTTGTTAATG 2339

OS ------ATATACATCGGGTACTGTGGCCCAATCCTTATTATACATTTTTGCTGAA--ACTAAATGCTCCAGTTGTTGGTG 935

Majority TCCTCTTTTTTA-------------------------TTCAGGTTCTTGACAATGGCATTCTTGTTGATCCCCACAATCA

---------+---------+---------+---------+---------+---------+---------+---------+

2410 2420 2430 2440 2450 2460 2470 2480

---------+---------+---------+---------+---------+---------+---------+---------+

TA(AA) TCCTCTTTTTTA-------------------------TTCAGGTTCTTGACAATGGCATTCTTGTTGATCCCCACAATCA 1116

TA(BB) TCCATTTTTTT---------------------------TCAGGTTCTTGACAATGGCATTCTTGTTGATCCCCACAATCA 976

AT TCCTCTTTTTTA-------------------------TTCAGGTTCTTGACAATGGCATTCTTGTTGATCCCCACAATCA 1015

HV TCCTCTTTTTTA-------------------------TTCAGGTTCTTGACAATGGCATTCTTGTTGATCCCCACAATCA 979

TU TCCTCTTTTTTA-------------------------TTCAGGTTCTTGACAATGGCATTCTTGTTGATCCCCACAATCA 1119

TS TCCTTTTTTTTTA------------------------TTCAGGTTCTTGACAATGGCATTCTTGTTGATCCCCACAATCA 992

BD TCATATATTTTAATGTTCTTTTGGTTTCATTTCTTCATTCAGGTTCTTGACAATGGTATTCTTGTTGATCCCCACAATCA 1468

SB TAGTATGTATTTTCCATGTTTTG-----ATTCCTTGGCTCAGGTTCTTGATAATGGAATTCTTGTTGACCCCCACAATCA 2414

OS TCATACATATTTCTCTTATTCTGATCATGTTTGTTTGTTCAGGTTCTTGACAATGGTATTCTCGTTGATCCCCACAATCA 1015

Majority AAATGATATAGCTGAGGCACTTTATAGACTTGTTTCTGATAAGCAATTGTGGGCAAAATGCCGTCAGAATGGTCTGGATA

---------+---------+---------+---------+---------+---------+---------+---------+

2490 2500 2510 2520 2530 2540 2550 2560

---------+---------+---------+---------+---------+---------+---------+---------+

TA(AA) AAATGATATAGCTGAGGCACTTTATAGACTTGTTTCTGATAAGCAATTGTGGGCAAAATGCCGTCAGAATGGTCTGGATA 1196

TA(BB) AAATGATATAGCTGAGGCACTTTATAGACTTGTTTCTGATAAGCAATTGTGGGCAAAATGCCGTCAGAATGGTCTGGATA 1056

AT AAATGATATAGCTGAGGCACTTTATAGACTTGTTTCTGACAAGCAATTGTGGGCAAAATGCCGTCAGAATGGTCTGGATA 1095

HV AAATGATATAGCTGAGGCACTTTATAGACTTGTTTCTGATAAGCAATTGTGGGCACAGTGCCGTAAGAATGGTCTGGAAA 1059

TU AAATGATATAGCTGAGGCACTTTATAGACTTGTTTCTGATAAGCAATTGTGGGCAAAATGCCGTCAGAATGGTCTGGATA 1199

TS AAATGATATAGCTGAGGCACTTTATAGACTTGTTTCTGATAAGCAATTGTGGGCAAAATGCCGTCAGAATGGTCTGGATA 1072

BD AAATGATATAGCTGAGGCACTTTATAAGCTTGTTTCTGATAAGCATTTGTGGGCAAAATGCCGTGAGAATGGTCTGCAAA 1548

SB AAATGAAATAGGTGAGGCACTTTATAAGCTTGTGTCAGATAAGCAATTGTGGACACGATGTCGCCAGAATGGTCTGAAAA 2494

OS AAATGAAATAGCTGAGGCACTTTATAAGCTTGTTTCTGATAAGCAGTTGTGGGCACAGTGCCGCCAAAATGGTCTGAAAA 1095

Majority ATATCCATCGATTTTCTTGGCCTGAACATTGCAAAAACTATTTGTCACGGGTTGGTACGCTCAAGTCTAGACATCCACGA

---------+---------+---------+---------+---------+---------+---------+---------+

2570 2580 2590 2600 2610 2620 2630 2640

---------+---------+---------+---------+---------+---------+---------+---------+

TA(AA) ATATCCATCGATTTTCTTGGCCTGAACATTGCAAAAACTATTTGTCACGGGTTGGTACGCTCAAGTCTAGACATCCACGA 1276

TA(BB) ATATCCATCGATTTTCTTGGCCTGAACATTGCAAAAACTATTTGTCACGGGTTGGTACGCTCAAGTCTAGACATCCACGA 1136

AT ATATCCATCGATTTTCTTGGCCTGAACATTGCAAGAACTATTTGTCACGGGTTGGTACGCTCAAGTCTAGACATCCACAA 1175

HV ATATCCATCGATTTTCTTGGCCTGAACATTGCAAGAACTATCTGTCACGGGTTGGTACGCTCAAGTCTAGACATCCACGA 1139

TU ATATCCATCGATTTTCTTGGCCTGAACATTGCAAAAACTATTTGTCACGGGTTGGTACGCTCAAGTCTAGACATCCACGA 1279

TS ATATCCATCGATTTTCTTGGCCTGAACATTGCAAAAACTATTTGTCACGGGTTGGTACGCTCAAGTCTAGACATCCACGT 1152

BD ATATCCATAGATTTTCTTGGCCAGAACATTGCAAGAACTATTTGTCACGGGTTGGTACACTTAAGCCTAGACACCCAAGA 1628

SB ACATCCATCAATTTTCGTGGCCTGAACATTGCAAGAACTATTTGGCACGTGTAGTCACTCTCAAGCCTAGACATCCCCGC 2574

OS ATATTCATCAATTTTCTTGGCCTGAACATTGCAAAAACTATTTGTCACGGGTTGGTACACTCAAGCCACGACATCCTCGA 1175

Majority TGGCAAAAGAGTGATGATGCTACTGAAGTTTCTGAAACAGATTCACCTGGTGACTCTTTGAGGGATATTCATGATATATC

---------+---------+---------+---------+---------+---------+---------+---------+

2650 2660 2670 2680 2690 2700 2710 2720

---------+---------+---------+---------+---------+---------+---------+---------+

TA(AA) TGGCAAAAGAGCGATGATGCTACTGAAGTTTCTGAAACAGATTCACGTGGTGACTCTTTGAGGGATATTCATGATATATC 1356

TA(BB) TGGCAAAAGAGCGATGATGCTACTGAAGTTTCTGAAACAGATTCACCTGGTGACTCTTTGAGGGATATTCATGATATATC 1216

AT TGGCAAAAGAGTGATGATGCTACTGAAGTTTCTGAAACTGATTCACCTGGTGACTCTTTGAGGGATATTCATGATATATC 1255

HV TGGCAAAGGAGTGATGATGCTACTGAAGTTTCTGAAACAGATTCACCTGGTGACTCTTTGAGGGATATTCATGATATATC 1219

TU TGGCAAAAGAGCGATGATGCTACTGAAGTTTCTGAAACAGATTCACCTGGTGACTCTTTGAGGGATATTCATGATATATC 1359

TS TGGCAAAAGAGCGATGATGCTACTGAAGTTTCTGAAACTGATTCACCTGGGGACTCTTTGAGGGATATTCATGATATATC 1232

BD TGGCAAAGGAGTGATGATGCTACTGAAATTTCTGAAGCAGATTCACCTGGCGACTCTTTGAGGGATCTTCATGATATATC 1708

SB TGGCAAAAGAATGATGTTGCAACTGAAATATCTGAAGCAGACTCACCTGAGGACTCTCTGAGGGATATCCATGACATATC 2654

OS TGGCAAAAGAGTGATGATGCTACTGAAGTTTCCGAAGCTGACTCACCTGGAGATTCCTTGAGGGATGTTCATGATATATC 1255

Majority ACTTAACTTGAAGATTTCCTTGGACAGTGAAAAATCAGGCAGCATGTCAAAATATGGAAGGAGTTCAACCAGTGATAGGA

---------+---------+---------+---------+---------+---------+---------+---------+

2730 2740 2750 2760 2770 2780 2790 2800

---------+---------+---------+---------+---------+---------+---------+---------+

TA(AA) ACTTAACTTGAAGATCTCCTTGGACAGTGAAAAATCAGGCAGCATGTCAAAATATGGAAGGAGTTCAACCAGTGACAGGA 1436

TA(BB) ACTTAACTTGAAGATTTCCTTGGACAGTGAAAAATCAGGCAACATGTCGAAATATGGAAGGAGTTCAACCAGTGATAGGA 1296

AT ACTTAACTTGAAGATTTCCTTGGACAGTGAAAAATCAGGCAGCATGTCAAAATATGGAAGGAGTTCAACCAGTGATAGGA 1335

HV TCTTAACTTAAAGATTTCCTTGGACAGTGAAAAATCAGGCAACATGTCAAAATATGGAAGGAGTTCAACCAATGAGAGGA 1299

TU ACTTAACTTGAAGATCTCCTTGGACAGTGAAAAATCAGGCAGCATGTCAAAATATGGAAGGAGTTCAACCAGTGATAGGA 1439

TS ACTTAACTTGAAGATTTCCTTGGACAGTGAAAAATCAGGCAACATGTCGAAATATGGAAGGAGTTCAACCAGTGATAGGA 1312

BD TCTTAACTTGAAGATTTCCTTAGACAGTGAAAAATCAAGCA-----CCA--------A--------------------GA 1755

SB ACTTAACTTGAAGCTTTCCTTGGACAGTGAAAAATCAGGCAG-----CAAA----GAAGGGAATTCAAATACTGTGAGAA 2725

OS TCTTAACTTGAAGCTTTCCTTGGACAGTGAGAAATCAAGCA------CAAA----GGAA--------AATAGTGTAAGAA 1317

Majority GAAACCTTGAGGATGCTGTACAAAAATTTTCAGAAGCTGTTAGTGCTGGCACAAAGGATGAGTCTGGTGAGAAAGCTGAG

---------+---------+---------+---------+---------+---------+---------+---------+

2810 2820 2830 2840 2850 2860 2870 2880

---------+---------+---------+---------+---------+---------+---------+---------+

TA(AA) GAAACCTTGAGGATGCTGTACAAAAATTTTCAGAAGCTGTTAGTGCTGGCACAAAGGATGAGTCTGGTGAGAAAGCTGGG 1516

TA(BB) GAAACCTTGAGGATGCTGTACAAAAGTTTTCAGAAGCTGTTAGTGCTGGCACAAAGGATGAGTCTGGTGAGAAAGCTGAG 1376

AT GAAACCTTGAGGATGCTGTACAAAAATTTTCAGAAGCTGTTAGTGCTGGCACAAAGGATGAGTCTGGTGAGAAAGCTGGG 1415

HV GAAACATTGAGGATGCTGTACTAAAATTTTCAGAAGCTGTTAGCGCTGGCACAAAAGATGAGTCTGGTGAGAATGCTGAG 1379

TU GAAACCTTGAGGATGCTGTACAAAAATTTTCAGAAGCTGTTAGTGCTGGCACAAAGGATGAGTCTGGTGAGAAAGCTGGG 1519

TS GAAACCTTGAGGATGCTGTACAAAAGTTTTCAGAAGCTGTTAGGGCTGGCACAAAGGATGAGTCTGGTGAGAAAGCTGAG 1392

BD GAAACCTTGAGGATGCTCTACTAAAATTTTCAAAAGGTGTTAGTGCCAGCACAAAGGATGAGTCTGGTGAGAATGCTGAG 1835

SB GGCATTTGGAGGATGCAGTGCAAAAGTTGTCA---GGTGTTAGTGACATCAAAAAGGATGGGCCAGGTGAGAAT-----G 2797

OS GAAACCTTGAGGATGCTGTACAAAAGTTGTCAAGAGGTGTTAGTGCCAACAGAAAGACAGAGTCTGTTGAGAATATGGAG 1397

Majority GCCACCACAGGCTCCAATAAATGGCCATCTCTGCGAAGGAGAAAGCACATCGTTGTTATTGCTGTAGATTCTGTGCAAGA

---------+---------+---------+---------+---------+---------+---------+---------+

2890 2900 2910 2920 2930 2940 2950 2960

---------+---------+---------+---------+---------+---------+---------+---------+

TA(AA) GCCACCACAGGCTCCAATAAATGGCCATCTCTGCGAAGGAGAAAACACATCGTTGTTATTGCTGTAGATTCTGTGCAAGA 1596

TA(BB) GCCACCACAGGCTCCAATAAATGGCCATCTCTGCGAAGGAGAAAGCACATTGTTGTTATTGCTGTAGATTCTGTGCAAGA 1456

AT GCCACCACAGGCTCCACTAAATGGCCATCTCTGCGAAGGAGAAAGCACATCGTTGTTATTGCTGTAGATTCTGTGCAAGA 1495

HV GCCACCACAGGCTCCAATAAATGGCCATCTCTGCGAAGGAGAAAGCACATCGTTGTTATTGCTGTAGATTCTGTGCAAGA 1459

TU GCCACCACAGGCTCCAATAAATGGCCATCTCTGCGAAGGAGAAAACACATCGTTGTTATTGCTGTAGATTCTGTGCAAGA 1599

TS GCCACCACAGGCTCCAATAAATGGCCATCTCTGCAAAGGAGAAAGCACATTGTTGTTATTGCTGTARATTCTGTGCAAGA 1472

BD GCTACCACAGGTTCCAATAAATGGCCATCTCTGCGAAGGAGAAAACACATTGTTGTGATAGCTGTAGATTCTGTGCAAGA 1915

SB ----------------GTAAGTGGCCATCATTGCGTAGGAGGAAGCACATCATTGTAATTGCTGTAGACTCTGTGCAAGA 2861

OS GCTACCACAGG--C-AATAAATGGCCATCTTTGCGAAGAAGGAAACACATTGTAGTCATTGCTATAGATTCTGTGCAAGA 1474

Majority TGCGGACTTGGTTCAGATTATCAAAAACATTTTTCAGGCTTCAAGCAAAGAAAAATCATCTGGTGCTCTTGGTTTTGTAT

---------+---------+---------+---------+---------+---------+---------+---------+

2970 2980 2990 3000 3010 3020 3030 3040

---------+---------+---------+---------+---------+---------+---------+---------+

TA(AA) TGCGGACTTGGTTCAGATTATCAAAAACATTTTTCAGGCTTCAAACAAAGAAAAATCATCTGGTGCTCTTGGTTTTGTAT 1676

TA(BB) TGCCGACTTGGTTCAGATTATCAAAAACATTTTTCAGGCTTCAAACAAAGAAAAATCATCTGGTGCTCTTGGTTTTGTAT 1536

AT TGCGGACTTGGTTCAGATTATCAAAAACATTTTTCAGGCTTCAAGCAAAGAAAAATCATCTGGCGCTCTTGGTTTTGTAT 1575

HV TGCGGACTTGGTTCAGATTATCAAAAACATTTTCCAGGCTTCAAGCAAAGAAAAATCATCTGGTGCTCTTGGTTTTGTAT 1539

TU TGCGGACTTGGTTCAGATTATCAAAAACATTTTTCAGGCTTCAAACAAAGAAAAATCATCTGGTGCTCTTGGTTTTGTAT 1679

TS TGCCGACTTGGTTCAGATTATCAAAAACATTTTTCAGGCTTCAAACAAAGAAAAATCATCTGGTGCTCTTGGTTTTGTAT 1552

BD TGCAGACTTAGTTCAGATTATAAAAAACATTTTTGAGGCTTCAAGCAAAGAAAGACTGTCCGGTGATGTTGGTTTTGTAT 1995

SB TGCAGACTTTGTTCAGGTTATTAAAAGTATTTTTGAGGCTTCAAGCAATGAGAGATCAAATGGCTCTGTTGGTTTTGTAT 2941

OS TGCTAACCTGGTTGAGATTATCAAAAATATTTTTGTGGCTTCAAGCAATGAGAGATTATCTGGTTCTGTTGGTTTTGTTT 1554

Majority TGTCAACATCTCGAGCAGCATCAGAGATACATCCTTTGTTAACATCTGGGGGCATAGAAATTACTGATTTTGATGCCTTC

---------+---------+---------+---------+---------+---------+---------+---------+

3050 3060 3070 3080 3090 3100 3110 3120

---------+---------+---------+---------+---------+---------+---------+---------+

TA(AA) TGTCAACATCTCGAGCAGCATCAGAGATACATCCTTTGTTAACATCTGGGGGCATAGAAATTACTGATTTTGATGCCTTC 1756

TA(BB) TGTCAACATCTCGAGCAGCATCAGAGATACATCCTTTGTTAACATCTGGGGGCATAGAAATTACTGATTTTGATGCCTTC 1616

AT TGTCAACATCTCGAGCAGCATCAGAGATACATCCTTTGTTAACATCTGGGGGCATAGAAATTACTGATTTTGATGCCTTC 1655

HV TGTCAACATCTCGAGCAGCATCAGAGATACATCCTTTGTTAACATCTGGGGGCATAGAGATTGCTGATTTTGATGCCTTC 1619

TU TGTCAACATCTCGAGCAGCATCAGAGATACATCCTTTGTTAACATCTGGGGGCATAGAAATTACTGATTTTGATGCCTTC 1759

TS TGTCAACATCTCGAGCAGCATCAGAGATACATCCTTTGTTAACATCTGGGGGCATAGAAATTACTGATTTTGATGCCTTC 1632

BD TGTCAACATCTCGAGCCGTATCAGAGATACATCCTTTGTTAACATCTGGGGGCATAGAAACTACTGATTTTGATGCCTTC 2075

SB TGTCAACGGCTAGAGCAATATCAGAGATACATGCTTTGCTTATATCTGGAGGGATAGAAGCTAGTGACTTTGACGCCTTC 3021

OS TGTCAACATCCCGAGCAATATCAGAGGTACATTCTTTGCTAACATCTGGGGGCATAGAAGCTACTGATTTTGATGCCTTC 1634

Majority ATATGCAGCAGTGGCAGTGATCTTTGCTATCCATCTTCAAATTCAGAAGACATGCTTAGCCCTGCCGAGCTTCCATTTAT

---------+---------+---------+---------+---------+---------+---------+---------+

3130 3140 3150 3160 3170 3180 3190 3200

---------+---------+---------+---------+---------+---------+---------+---------+

TA(AA) ATATGCAGCAGTGGCAGTGATCTTTGCTATCCATCTTCAAATTCAGAAGACATGCTTAGCCCTGCCGAGCTTCCATTTAT 1836

TA(BB) ATATGTAGCAGTGGCAGTGATCTTTGCTATCCATCTTCAAATTCAGAAGACATGCTTAGCCCTGCCGAGCTTCCATTTAT 1696

AT ATATGCAGCAGTGGCAGTGATCTTTGCTATCCATCTTCAAATTCAGAAGACATGCTTAGCCCTGCCGAGCTTCCGTTTAT 1735

HV ATATGCAGCAGTGGCAGTGATCTTTGCTATCCATCTTCAAATTCAGAAGACATGCTTAGCCCTGCCGAGCTTCCATTTAT 1699

TU ATATGCAGCAGTGGCAGTGATCTTTGCTATCCATCTTCAAATTCAGAAGACATGCTTAGCCCTGCCGAGCTTCCATTTAT 1839

TS ATAGGCAGCAGTGGCAGTGATCTTTGCTATCCATCTTCAAATTCAGAAGACATGCTTAGCCCTGCCGAGCTTCCATTTAT 1712

BD ATATGCAGCAGTGGCAGTGATCTTTGCTATCCGTCTGCAAGTTCTGAAGACATGCTTAGCCCTTCCGAGCTTCCATTTAT 2155

SB ATATGCAACAGTGGCAGTGATCTTTGTTATCCATCTTCAAACTCTGAGGACATGCTTAGCCCTGCTGAGCTCCCATTCAT 3101

OS ATATGCAACAGTGGTAGCGATCTTTGCTATCCATCCTCAAATTCTGAAGACATGCTTAGCCCTGCAGAGCTCCCATTTAT 1714

Majority GATCGATCTTGATTATCACTCTCAGATTCAATATCGTTGGGGAGGAGAAGGTTTAAGGAAGACACTAATTCGTTGGGCAG

---------+---------+---------+---------+---------+---------+---------+---------+

3210 3220 3230 3240 3250 3260 3270 3280

---------+---------+---------+---------+---------+---------+---------+---------+

TA(AA) GATCGATCTTGATTATCACTCTCAGATTCAATATCGTTGGGGAGGAGAAGGTTTAAGGAAGACACTAATTCGTTGGGCAG 1916

TA(BB) GATCGATCTTGATTATCACTCTCAGATTCAATATCGTTGGGGAGGAGAAGGTTTAAGGAAGACACTAATTCGTTGGGCAG 1776

AT GATCGATCTTGATTATCACTCTCAGATTCAATATCGTTGGGGAGGAGAAGGTTTAAGGAAGACACTGATTCGTTGGGCGG 1815

HV GATCGATCTTGATTATCACTCTCAGATTCAATATCGCTGGGGAGGAGAAGGTTTAAGGAAGACACTAATTCGTTGGGCAG 1779

TU GATCGATCTTGATTATCACTCTCAGATTCAATATCGTTGGGGAGGAGAAGGTTTAAGGAAGACACTAATTCGTTGGGCAG 1919

TS GATTGATCTTGATTATCACTCTCAGATTCAATATCGTTGGGGAGGAGAAGGTTTAAGGAAGACACTAATTCGCTGGGCAG 1792

BD GATTGATCTTGATTATCACTCTCAAATTGAATATCGCTGGGGAGGAGAAGGTTTAAGGAAGACACTAATTCGTTGGGCAG 2235

SB GATTGATCTTGATTATCACTCCCAAATTGAATATCGCTGGGGAGGAGAAGGTTTAAGGAAGACACTAATTCGTTGGGCGT 3181

OS GATTGATCTTGATTATCACACTCAAATTGAGTACCGTTGGGGTGGAGAAGGTTTAAGGAAGACACTAATTTGTTGGGCAG 1794

Majority CAGAAAAGAATAGCGAGAGTGGACAAGAAGCAGTTGTTGAAGATGACGAATGTTCATCCACTTACTGCATTTCATTTAAA

---------+---------+---------+---------+---------+---------+---------+---------+

3290 3300 3310 3320 3330 3340 3350 3360

---------+---------+---------+---------+---------+---------+---------+---------+

TA(AA) CAGAAAAGAATAGCGAGAGTGGAAAAGAAGCAGTTGTTGAAGATGACGAATGTTCATCCACTTACTGCATTTCATTTAAA 1996

TA(BB) CAGAAAAGAACAGCGAGAGTGGACAAGAAGCAGTTGTCGAAGATGACGAATGTTCATCCACTTACTGCATTTCATTTAAA 1856

AT CAGAAAAGAATAGCGAGAGTGGACAAGAAGCAGTTGTTGAAGATGACGAATGTTCATCCACTTACTGCATTTCATTTAAA 1895

HV CAGAAAAGAATAGCGAGAGGGGACAAGAAGCAGTTACTGAAGATGACGAATGTTCATCCACTTACTGCATTTCTTTTAAA 1859

TU CAGAAAAGAATAGCGAGAGTGGAAAAGAAGCAGTTGTTGAAGATGACGAATGTTCATCCACTTACTGCATTTCATTTAAA 1999

TS CAGAAAAGAACAGTGAGAGTGGACAAGAAGCAGTTGTTGAAGATGATGAATGTTCATCCACTTACTGCATTTCATTTAAA 1872

BD CAGAAAAGAACAGTGAGAGTGGACAAAAAGTCGTCGTTGAAGATGAAGAATGTTCGTCAACTTACTGCATTTCATTTAAA 2315

SB CTGAGAAAAATAATGAAAGTGGACAAAAAATACTTGTTGAGGATGAAGAATGCTCATCCACTTACTGCATTTCATTTAAA 3261

OS CAGAAAAAA---GCGAGGGTGGTCAAGTGGTACTTGTAGAAGATGAAGAATGTTCATCCACTTATTGTATTTCATTTAGA 1871

Majority GTGAAGAATACTGAGGCTGTACGTTTGTTCTTCGTCCTGTTATGCCCTCTTCATCTACTTCTTCACTGATTTTCTCA-AC

---------+---------+---------+---------+---------+---------+---------+---------+

3370 3380 3390 3400 3410 3420 3430 3440

---------+---------+---------+---------+---------+---------+---------+---------+

TA(AA) GTGAAGAATACTGAGGCTGTACGTTTGTTCTTCGTCCTGTTATGCCCTCTTCATCTACTTCTTCACTGATTTTCTCA-AC 2075

TA(BB) GTGAAGAATACTGAGGCTGTACGTTTGTTCTTCGTCCTGTTATTCCCTCTTCATCTACTT---CACAGATTTT--CA-AC 1930

AT GTGAAGAATACTGAGGCTGTACGTTTGTTCTTCATCCTGTTATGCCCTCTTCATCTACTTCTTCACTGATTTT------C 1969

HV GTGAAGAATACTGAGGCCGTACGTTTGTTCTTC-------------------ATCTACTTCTTCACTGATTTTCTCA-AC 1919

TU GTGAAGAATACTGAGGCTGTACGTTTGTTCTTCGTCCTGTTATGCCCTCTTCATCTACTTCTTCACTGATTTTCTCA-AC 2078

TS GTGAAGAATACCGAGGCCGTACGTTTCTTCTTCGTCCCGTTATGCCCTCTTCATCTACTA---AACTGATTTTCTCA-AC 1948

BD GTGAAGAATTCTGAGGCTGTATGTTTCTTCTTCATCCTTCTATGCCCTCTTCATTTATGTTTTCAATGATTGTCTTT-AC 2394

SB GTGTCCAATACTGCAGCTGTATGTTTCTTAGTC---TTGTTGTGCTCT----GTCAAGTTATCCACTGACTTTTTTAAAT 3334

OS GTGAAGAATGCTGAGGCTGTATGTTTCTT-TTCGTCCTGTTAGCCACTTCTCATAGCCTT-----CTGATTTTCTCGGAC 1945

Majority CTTCTCTACT-ATAGCCGGTGTTCTTATTATGCTTCAACTTTCAACTGTTTTAGGTCCCTCCTGTGAAGGATCTTAGGAA

---------+---------+---------+---------+---------+---------+---------+---------+

3450 3460 3470 3480 3490 3500 3510 3520

---------+---------+---------+---------+---------+---------+---------+---------+

TA(AA) CTTCCCTACT-ATAGCCGGTGTTCTTATTATGCTTCAACTTTCAACTGTTTTAGGTCCCTCCTGTGAAGGATCTTAGGAA 2154

TA(BB) CTTCTCTACT-ATGGCCAGTGTTCTTATTATGCTTCAACTTTCAACTGTTTTAGGTCCCTCCTGTGAAGGATCTTAGGAA 2009

AT CTTCTCTACT-ATAGCCAATGTTCTTATTATGCTTCAACTTTCAACTGTTTTAGGTCCCTCCTGTGAAGGATCTTAGGAA 2048

HV CTTCTCTACT-ATAGACGGTGTTCTTATTATGCTTCAACTTTCATATGTTTTAGGTCCCTCCTGTGAAGGATCTTAGAAA 1998

TU CTTCCCTACT-ATAGCCGGTGTTCTTATTATGCTTCAACTTTCAACTGTTTTAGGTCCCTCCTGTGAAGGATCTTAGGAA 2157

TS GTTCTCTACT-ATAGCCGGTGTTCTTATTATGCTTCAACTTTCAACTGTTTTAGGTCCCTCCTGTGAAGGATCTTAGAAA 2027

BD ATTCTGTACA-CTCGTTGGTGTT-TTATTATG-------TTGCAACTGTTTTAGGTCCCTCCGGTGAAAGACCTTAGAAA 2465

SB ATCTTCTATACATTATTGATGAG-ATAATATG-------TTTCACTTGTTT-AGGCACCTCCTGTGAAGGAGATTAGGAG 3405

OS ATTTTGTAAG-TTACCTGATGTT----TTATG-----ATTTTCATGTGTTTCAGGTACCTCCTGTGAAAGAGCTTAGGAA 2015

Majority GACAATGAGAATTCAAGCATTGCGCTGTCA-TGTATTGTACAGCCATGATGGCAGCAAGTTGAACTTCATACCTGTTCTA

---------+---------+---------+---------+---------+---------+---------+---------+

3530 3540 3550 3560 3570 3580 3590 3600

---------+---------+---------+---------+---------+---------+---------+---------+

TA(AA) GACAATGAGAATTCAAGCATTGCGCTGTCA-TGTATTGTACAGCCATGATGGCAGCAAGTTGAACTTCATACCTGTTCTA 2233

TA(BB) GACAATGAGAATTCAAGCATTGCGCTGTCA-TGTATTGTACAGCCATGATGGCAGCAAGTTGAACTTCATACCTGTTCTA 2088

AT GACAATGAGAATTCAAGCATTGCGCTGTCA-TGTATTGTACAGCCATGATGGCAGCAAGTTGAACTTCATACCTGTTCTA 2127

HV GACAATGAGAATTCAAGCATTGCGGTGTCA-TGTATTGTACAGCCATGATGGCAGCAAGTTGAACTTCATACCTGTTCTA 2077

TU GACAATGAGAATTCAAGCATTGCGCTGTCAATGTATTGTACAGCCATGATGGCAGCAAGTTGAACTTCATACCTGTTCTA 2237

TS GACAATGAGAATTCAAGCATTGCGCTGTCA-TGTATTGTACAGCCATGATGGTAGCAAGTTGAACTTCATACCTGTTCTA 2106

BD GACAATGAGAATTCAAGCTCTACGCTGTCA-TGTTTTGTACAGCCATGATGGTAGCAAATTGAATCTTATTCCTGTCCTA 2544

SB GACAATGAGAATTCAAGCACTGCGCTGCCA-TGTTTTGTACAGCCATGATGGTAGCAAGCTGAACGTAATTCCTGTTTTG 3484

OS GACAATGAGAATTCAAGCACTGCGCTGTCA-TGTTTTGTACAGTCATGATGGTAGCAAGTTGAATGTTATTCCTGTTTTA 2094

Majority GCATCACGATCCCAAGCACTAAGGTTT-GTTTATTCCTATAACTGTTXTATATAT-TTTTTGAACACAGCCTTCAGTCCT

---------+---------+---------+---------+---------+---------+---------+---------+

3610 3620 3630 3640 3650 3660 3670 3680

---------+---------+---------+---------+---------+---------+---------+---------+

TA(AA) GCATCACGATCCCAAGCACTAAGGTTT-GTTTATTCCTATAAATGCTGTATATAT-TTTCTGAACACAGCCTTGAGTCCA 2311

TA(BB) GCATCACGATCCCAAGCGCTAAGGTTT-GGTTATTCCTATAACTGTTATATATAT-ATTTTGAACACAGCCTTCAGTCCT 2166

AT GCATCACGATCCCAAGCGCTAAGGTTT-GTTTATTCATATAACTGTTGTATATAT-TTTTTGAACACAGCCTTCAGTCCT 2205

HV GCATCACGATCCCAAGCAATAAAGTTTTGTTTATTCATATAACTGTTATATATAC-TTTTTGAACACAACCTTCAGTCCT 2156

TU GCATCACGATCCCAAGCACTAAGGTTT-GTTTATTCCTATAAATGCTGTATATAT-TTTCTGAACACAGCCTTCAGTCCA 2315

TS GCATCACGATCCCAAGCACTAAGGTTT-GGTTATTCCTATAACTGTTATATATAT-ATTTTGAACACAGCCTTCAATCCT 2184

BD GCATCACGATCACAAGCACTAAGGTTT-GACTACTCCTATAACTGTTCTCTTT---TTCATGGGCAGAGCCTTCAAACCT 2620

SB GCTTCTCGCTCACAGGCTTTAAGGTCT-GATTGTTACTTTAATGCTTCCCTTTCTTTCCACAAGCTTGTTCCTTAATTTG 3563

OS GCATCACGATCGCAGGCTCTAAGGTCGACTTTCTTTGTTCAACTGCTCT-TCCAT-TTTTTCTCCTCATGTTGCCATGTT 2172

Majority CAG---TTGAGTAAAATGCTATACT-----TTGTACGG--------TTA--TAATCTAT-CTGCCACTAC-ATGTTAACC

---------+---------+---------+---------+---------+---------+---------+---------+

3690 3700 3710 3720 3730 3740 3750 3760

---------+---------+---------+---------+---------+---------+---------+---------+

TA(AA) CAG---TTGAGTAAAATGCTATACT-----TTGTACGG--------TTA--TAATCTAT-CTGCCACTAC-ATGTTAACC 2371

TA(BB) CAG---TTGAGTAAAA-------CT-----TTTTACGG--------TTA--TAATCTAT-CTGCCACTAC-ATGTTAACC 2219

AT CAG---TTGAGTAAAATGCTATACT-----TTGTATGG--------TTA--TAATCTAT-CTGCCACTAC-ATGTTAACC 2265

HV CTG---TTCACTATAATGCTATACT-----TTGTATGA--------TTA--TACTCTAT-CTGCCACTAC-ATGTTAACC 2216

TU CAG---TTGAGTAAAATGCTATACT-----TTGTACGG--------TTA--TAATCTAT-CTGCCACTAC-ATGTTAACC 2375

TS CAG---TTGATATAAATGCTATACT-----TTGTACGG--------TTA--TAATCTAT-CTGCCACTAC-ATGTTAACC 2244

BD CTAGTCTCGAGTAAAATG------------TTATACTG--------TTA--CTCTCTCTGCTGCCACTA--ACGGTTGCC 2676

SB CATAAATTGTGGGTTGCCCTTGAACTTCCACAGTAATGTCCAAGGTTTGACCAACTTGTAATGTGGTCATCATGTGAATT 3643

OS CCTCATTGGAGCAAAATATTATACTCAGTTTTCCACTAAATAAAGTTTA--CATATAATTTTCATTCCGC-AAAGTAAAC 2249

Majority X-------TTCTAGGATGCTTGTA-GTTCTGATCATCAA-TTTG----TATGAAGT-----------------GTTTTTG

---------+---------+---------+---------+---------+---------+---------+---------+

3770 3780 3790 3800 3810 3820 3830 3840

---------+---------+---------+---------+---------+---------+---------+---------+

TA(AA) G-------TTCTAGGATGTTTGTA-GTTCTGATCATCAA-TTTG----TATGAAGT-----------------GTTTTTG 2421

TA(BB) --------TTCTAGGATGCTTGT-------GATCATCAA-TTTG----TATGAAGT-----------------GTTTTTG 2262

AT --------TTCTAGGATGCTTGTA-GTTCTGATCATCAA-TTTG----TATGAAGT-----------------GTTTTTG 2314

HV G-------TTCTAGGATGCTTGTA-ATTCTGATCATCAA-TTTG----TATGAAGT-----------------GTTTTTG 2266

TU G-------TTCTAGGATGTTTGTA-GTTCTGATCATCAA-TTTG----TATGAAGT-----------------GTTTTTA 2425

TS --------TTCTAGGATGCTTGTA-GTTCTGATCATCAA-TTTG----TATGAAGT-----------------GTTTTTG 2293

BD A-------TTCTAGAATGCTTGTA-AT--TGACCATCA--TTTG----TAGGAAGTATTTTTGTT-TT----TGTTTTTG 2735

SB TG--AAATTAGTAGATTTATTATATATAATACTCCCTCTGTTCCAAATTATAAAATGGTTTGGCTTTTAGATTGCTTTTA 3721

OS AAGTAAAGACTTAGATAACATATA-TTTTTTATTGCTATATTTGGTCATATATACTCCCATCAATATT----TATTTTTG 2324

Majority CGAGG-------TAACAGAGT-AT-----------------ATAGATTGATCGC-ATATATTACAATXGAAAA------T

---------+---------+---------+---------+---------+---------+---------+---------+

3850 3860 3870 3880 3890 3900 3910 3920

---------+---------+---------+---------+---------+---------+---------+---------+

TA(AA) CGAGG-------TAACAGAGT-AT-----------------ATAGATTGATCGC-ATATATTACAATAGAAAA------T 2469

TA(BB) CGAGG-------TAACAGAGT-AT-----------------ATAGATTGATTGC-ATATATTACAATCGAAAA------T 2310

AT CGAGG-------TAACAGAGT-AT-----------------ATAGATTGATCAC-ATATATTACGATCGAAA-------T 2361

HV CGAGG-------TAACAAAGT-AT-----------------ATAGATTGATGGC-ATATATTACAATCGAAAA------T 2314

TU CGAGG-------TAACAGAGT-AT-----------------ATAGATTGATCGC-ATATATTACAATAGAAAA------T 2473

TS CGAGG-------TAACAGAGT-AT-----------------ATAGATTGATCGC-ATATATTACAATCGAAA-------T 2340

BD CAAGG-------TAACAAAAT-ACTGA--------------GTATATAGATTGA-ATGGACTCCTATTTTGTA------C 2786

SB CTACTATGTATCTAGAAAAGCCAAACATCTTATAATTTGTAATGGAGCGATTATTCCATAACAAAATAATATATAGTTTT 3801

OS TGAGTGT-----TAACATCAT-ATACTCAGTTTCCAATTCTAAAGTTTGATCACTGTCTTTCCTGATAATAAAT--CCAT 2396

Majority AGCGGTCATA-GTAGAACATCGTTATGTCAAT--GTCCTGAATG---ACTTATATTATXCTCTGATACTAGAA--TACAT

---------+---------+---------+---------+---------+---------+---------+---------+

3930 3940 3950 3960 3970 3980 3990 4000

---------+---------+---------+---------+---------+---------+---------+---------+

TA(AA) AGCGGTCATA-GTAGAACATCGTTATATCAGT--GTTCTGAATG---ACTTATATTATACTCTGATACTAGAA--TACAT 2541

TA(BB) AGCGGTCATA-GTAGAACATCGTTATGTCAAT--GTCCTGAATG---ACTTGTATTATGCTCTGATACTCGAA--TACAT 2382

AT AGTGGTCATA-GTAGGACATCGTTATGTCAGT--GTCCTGAATG---ACTT----------------------------- 2406

HV AGTGGTCATA-GTAGAACATCGTTAGGTCAAT--GTCCTGAATG---ACTTATATTATACTCTGATACTGGAA--TACAT 2386

TU AGCGGTCATA-GTAGAACATCGTTATATCAAT--GTTCTGAATG---ACTTATATTATACTCTGATACTAGAA--TACAT 2545

TS AGCGGTCATA-TTAGAGCATCGTTATGTCAGT--GTCCTGAATG---ACT--T--------------------------- 2385

BD TTCGAGGAGT-ATGTACTCCCATCTTTTGACA--CTGATTCATG---ATATTCCCTCCGTCCCATATTAAGTG--ACTCA 2858

SB AATGATTACAAACATTGTATTATAGTTGAAATCAGTGCTGGAAGTAATATCGAAGAGTGTTTGATGTCCAAATGTCACAC 3881

OS AAAAATTAAATGTATAACTACATGAACGTATT--TTTATGAAAATGTACTAATCATTTTTTTCATGTATAAAA--TCTAA 2472

Majority ATTTGATGTTGAAATGACXTTATTCATTGTTTAATAAATG---TAAAGAT------ATA------XXTAGXAXT-XGXCA

---------+---------+---------+---------+---------+---------+---------+---------+

4010 4020 4030 4040 4050 4060 4070 4080

---------+---------+---------+---------+---------+---------+---------+---------+

TA(AA) ATTTGATGTTGAAATGACGTTATTCATTGTTTAATAAATG---TGAAGAT------ATAGTAGAAGTTAGTAATGTGCCA 2612

TA(BB) ATTTCATGTTGAAATGACTATATTCATTGTTTAGTAAATG---TAAAGAT------ATA--------------------- 2432

AT ---------------------------------------------------------TA--------------------- 2408

HV ATTTGATGTTGGAATGACATTATTCATTGTTTAATATATG---TAAAGAT------GTACTCCCTTTGTAAACTAATGTA 2457

TU ATTTGATGTTGAAATGACATTATTCATTGTTTAATAAATG---TAAAGAT------ATAGTAGAAGTTAGTAATGTGCCA 2616

TS ---------------------------------------------------------TA--------------------- 2387

BD AATTTGTCTAAATATGGACGTATCTATATACTAAAATACG---TCTAGAT------ACA------TGTAATATTCCGGCA 2923

SB ATTTGGCCCCACCGATGATGTGCCGGTGACATGAGCTAGAACAACCAGATCGAGATGTAAGGAACGGGAGAAGATCTCCT 3961

OS CCTTTTTGCATATAGTAGCAAGTTAAGATTTTGAGGATTAGCCTAAAGAGCGAAA-ATTAATCTTAGTGGAAATCTGTCA 2551

Majority ----CX-T-XTXXCTCTGTTC--XXAAXATAATX-TTTXTAGTX-X-TXAGAAX-GACTATXTATTAATGTATXT--AGX

---------+---------+---------+---------+---------+---------+---------+---------+

4090 4100 4110 4120 4130 4140 4150 4160

---------+---------+---------+---------+---------+---------+---------+---------+

TA(AA) TGCACAGTACTCCCTCTGTTTC-TAAATATAAGTCTTTTTAGAGATTTCAGTAGGGACTATATACGGATGTATAT--AGA 2689

TA(BB) ---------CTCCCTCTGT------AA-ACTAA--------AT------------GATCTTATATTAGTTTACAG--AGG 2474

AT -------------------------------------------------------------------------------- 2408

HV AGATCATTTAGATCACTAAACG-ATCTTATATTAGTTTACAGAGGGAGTAGAAGTTAGTAATGCGCCATGCTTTT--ATT 2534

TU TGCACAGTACTCCCTCTGTTTC-TAAATATAAGTCTTTTTAGAGATTTCAGTAGGGACTATATACGGATGTATAT--AGA 2693

TS -------------------------------------------------------------------------------- 2387

BD ----C--T--TAATATGGGACG-GAGGGAGTACATCTTTCATTTTCAGTATACTTCAATAATGATTAAAAGATGT--ACT 2992

SB TTACCCATGATATTTTT--TCAGGTAGCAAATTTGTTCATATTTCCCTTATAGACGCCTGGTCTTTAA-GTACTCACACC 4038

OS CAAACAATATTTTGTTTGTTCTTGTAAGAGCATGTATTGTAGTCCCGTCTGAACATGCAAGCCATTATTGACTTTTCATG 2631

Majority XGTXXTA-AXXGAAGAXTCAXTXAAX-X-XX--------X-TTXATAATG-AX-CXXXXXX-----GXCATATATXTX-X

---------+---------+---------+---------+---------+---------+---------+---------+

4170 4180 4190 4200 4210 4220 4230 4240

---------+---------+---------+---------+---------+---------+---------+---------+

TA(AA) CGTATTATAGTGTAGATTCACTCATTTTGCTCCGTATGTAGTTCATAGTGGAATCTTTAAAAA---GACTTATATTTAGG 2766

TA(BB) -----GA----GTAGA-----------------------AGTTAGTAATG------C---------GCCATACA--C--- 2502

AT -------------------------------------------------------------------------------- 2408

HV CGTTTAAGAGGCATGGATGGCTGAAGAAACT----------TTAATAATCTGGTTCCC--------AATACAAACACAAG 2596

TU CGTATTAGAGTGTAGATTCACTCATTTTTCTCCGTATGTAGTTCATACTGGAATCTTTAAAAA---GACTTATATTTAGG 2770

TS -------------------------------------------------------------------------------- 2387

BD AATTGCAAGAAAAAATACCATTGGAACCC------------TTGGTTATGTA--CATAAAAAA---GCAGTATATATGAA 3055

SB AGCACTACACATAGACCCCAACCAATGGAAC--ATCAACAACTCATGATACAAGCTCACAA-----GGCGTAATTGCTTC 4111

OS GTCCCCAATCAAAGACTTTGACAAAAGTTCTGAATTGTGCTTTACCAGTAAAAATAATAGGATTTTGCTAGACAATTTTA 2711

Majority AXX-XAXXXATTXTTTTTTACT-------------XGTTTAAGAGGCATXGATTGCTGGAGAAAXTTTAATAATCTGGTT

---------+---------+---------+---------+---------+---------+---------+---------+

4250 4260 4270 4280 4290 4300 4310 4320

---------+---------+---------+---------+---------+---------+---------+---------+

TA(AA) AATGGAGGGAGTATTTTTTATT-------------CGTTTAAGAGGCATGGATTGCTGGAGAAACTTTAATAATCTGGTT 2833

TA(BB) ---------AATATTTTTTATT-------------CGTTTAAGAGGCATGGATTGCTGGGGAAACTTTAATAATCTGGTT 2560

AT ---------------------------------------------------------------------ATAATCTGGTT 2419

HV AGAAGTAAGATGTATGTAGGCG-------------TGTTCTACAAACACTGATTAGCTGATAAAAAAT-ACTCCCTCCGT 2662

TU AATGGAGGGAGTATTTTTTATT-------------CGTTTAAGAGGCATGGATTGCTGGAGAAACTTTAATAATCTGGTT 2837

TS ---------------------------------------------------------------------ATAATCTGGTT 2398

BD AATTAAGTTAAACATATGTACT-------------GAAATAAGAAGT-TTCATGG--GAGTCACTTTGGAGTGTCTAGTT 3119

SB ACACTGCTAGTTCCCGCGTCCTGAG-CGACCCTCTGTTGTGACCTCTATCC-CGCCTGCAGAGCTTGCCTGTCCCTGAGC 4189

OS GTACTATTTGTTCTCATATACTCCCTCCGTTTCAGGTTGTAAGACATTTTGACTTTGGTCAAAGTTAAACTGCTTTAAGT 2791

Majority CCCAATA----CAAA----CATAAGAGAAGTAAGATGT-------ATGT--AGGCGTGTTCTGCAAGCA--CCG------

---------+---------+---------+---------+---------+---------+---------+---------+

4330 4340 4350 4360 4370 4380 4390 4400

---------+---------+---------+---------+---------+---------+---------+---------+

TA(AA) CCCAATA----CAAA----CATAAGAGAAGTAAGATGT-------ATGT--AGGCGTGTTCTGCAAGCA--CCC------ 2888

TA(BB) CCCAATA----CAAA----CATAAGAGAAGTAAGATGT-------ATGT--AGGCGTGTTCTGCAAGCA--CCG------ 2615

AT CCTGGTA----CGAA----CATAAGAGCAGTAAGATGT-------ATGT--AGCCGTGTTCTGCAAGCA--CCG------ 2474

HV CCCATAA----TA------TAAAAGCGGCTTTTACACT-------ACAC--TAGTGAAATAAACGATCT--TAT------ 2715

TU CCCAATA----CAAA----CATAAGAGAAGTAAGATGT-------ATGT--AGGCGTGTTCTGCAAGCA--CCG------ 2892

TS CCTAATA----CA------CATAAGAGAAGTAAGATGT-------ATGT--TGGCATGTTCTGCAAGCA--CCG------ 2451

BD ATTACTAT--TCGAG----TATATTCAAAGTGATAAAG-------GAGTG-TGACACATCTCATAATTA--CTTTTT-TC 3182

SB AAGGATGGACTGAAACCTCTGTTGCAGGACATAGAGGTCTTCCCAGGTTGCTGACACCTCTGGCAGAGAAGCCCACTTGA 4269

OS TTGACCACGTTTATA----GAAAAAAGTAGTAACATTTTCAACCCAAGAT-AAATTTATTTTGAAAATA--TATTCAATT 2864

Majority ATTG------TCTGACAAAATC--TATTA--AGCATCAGAAGGCTGAAGGCTAGCAAGGGCGAGACCT---GTGTTTGGG

---------+---------+---------+---------+---------+---------+---------+---------+

4410 4420 4430 4440 4450 4460 4470 4480

---------+---------+---------+---------+---------+---------+---------+---------+

TA(AA) ATTG------GCTGACAAAATC--TATTA--AGCATCAGAAGGCTGAAGGCTAGCAAGGGCGAGACCT---GTGTTTGGG 2955

TA(BB) ATTG------TCTGATAAAATC--TATTA--AGCATCAGAAGGCTGAAGGCTAGCAAGGGCGAGACCT---GTGTTTGGG 2682

AT ATTG------GCTGACAAAACC--TATTA--AGCATCAGAAGGCTGAAGGCTAGCAAGGGTGAGACCT---GTGTTTGGG 2541

HV GTTA------TGGGACGGAGGGAGTATTA--AGCATCAGAAGGCTGAAGGCTAGCAAGGGCGAGACCT---GTGTCTGGG 2784

TU ATTG------GCTGACAAAATC--TATTA--AGCATCAGAAGGCTGAAGGCTAGCAAGGGCGAGACCT---GTGTTTGGG 2959

TS ATTG------TCTGACAAAATC--CATTA--AGCATCAGAAGGCTGAAGGCTAGCAAGGGCGAGGCCT---GTGTTTGGG 2518

BD ATTT------TGGTACTACATCATTATAA--AACTTCCATAGATTTATTTTTATTTATAACAGAATGTTG-GTAGCTTAT 3253

SB CCTACACTTGTGGAATTGCTTCAGTACCT--ATCTTGATGAGGCGCCGGTTGAGAATCGCTTCTGGTTGCAATGGTGTGG 4347

OS ATTGATTTGATGAAACTAATTTAGTATTATAAATATTACTATATTTGTCTATAAACTTAGTCAAACTTGAAACAGTTTGA 2944

Majority CTTTGG--ACCACAGTTAGATTXAGGCTTTCCT-----TTAGTGTTCTTTTTTXCTTCT---TTCCTGTAGGGTC-GTTT

---------+---------+---------+---------+---------+---------+---------+---------+

4490 4500 4510 4520 4530 4540 4550 4560

---------+---------+---------+---------+---------+---------+---------+---------+

TA(AA) CTTTGG--ACCACAGTTAGATTTAGGCTTTCCT-----TTAGTGTTCTTTTTTTCTTCT---TTCCTGTAGGGTC-GTTT 3024

TA(BB) CTTTGG--ACCACAGTTAGATTAAGGCTTTCCT-----TTAGTGTTCTTTTTTGGTTCT---TTCCTGTAGGGTC-GTTT 2751

AT CTTTGG--ACCACGGTTAGGTTTAGGCTTTCCT-----TTAGCGTTCTTTTTT-CTTCT---TTCCTATAGGGTCAGTTT 2610

HV CTTTGG--ACCACAGTTAGATTCAGGCTTTCCT-----TTAGTGTTATTTTTT-CTTAT---TTCCTGTAGGGTC-GTTT 2852

TU CTTTGG--ACCACAGTTAGATTTAGGCTTTCCT-----TTAGTGTTCTTTTTTTCTTCT---TTCCTGTAGGGTC-GTTT 3028

TS CTTTGG--ACCACAGTTAGATTTAGGCTTTCCT-----TTAGTGTTCTTTTTC-CTTCT---TTCCTGTTGGGTC-GTTT 2586

BD CATAGAGAATAGCAATCAGAATAGCACATTGGTGTGCGTTGATGTCTCAGATGACCTATATGTCACTCCTATACTAGAGT 3333

SB CTTCAAGATCAACCGCTTA--CAGCAACTCAGAATACACAGGTATAGTCCTGTTCTTGACAACCGTTCACCCATCCAGGC 4425

OS CTTTGACTGAAACGGAGGGAGTAGTCAATTTATATG-GTCGAGATACATGAATGATTGAA-GTTTTAGGATCGTGAGTGC 3022

Majority GACACCTC----ATTGTTGTATC----TAGACTGCTTTCCTA---CCTAATGAGAAGGTGXGA-TATGCTCTTTCA----

---------+---------+---------+---------+---------+---------+---------+---------+

4570 4580 4590 4600 4610 4620 4630 4640

---------+---------+---------+---------+---------+---------+---------+---------+

TA(AA) GACACCTC----ATTGTTGTATC----TAGACTACTTTCCTA---CCTAATGAGAAGGTGCGA-TATGCTCTTTCA---- 3088

TA(BB) GACACCTC----ATTGTTGTATC----TAGACTACTTTCCTA---CCTAATGAGAAGGTGCG--TATGCTCTTTCA---- 2814

AT GACACCTC----ATTGTTGTATCATTCTAGACTGCTTTCCTA---CCTAATGAGAAGATGTGA-TATGCTCTTTCA---- 2678

HV GACACCTC----ATTGTT--------------TGCTTTCCTA---CCTAATGAGAAGATGTGA-TATTCTCTTCAA---- 2906

TU GACACCTC----ATTGTTGTATC----TAGACTACTTTCCTA---CCTAATGAGAAGGTGCGA-TATGCTCTTTCA---- 3092

TS GACACCTC----ATTGTTCTATA----TAGACTGCTTTCCTA---CCTAATAAGAAGATGCGA-TATGCTCTTTCA---- 2650

BD ACAGCTTG----ATTGTTGAAATGATGTTAATTTTTTCCACGAGGCCAACTTTTCTTGCATGA-TGTCCAAAATTA---- 3404

SB ACGATTGTCA--AGAGTTTGAGCA---ACAGTCCTGTTCTTGACAACCGTTCTGGAGTCTTCT-TGTGCAAGTTTGGAGC 4499

OS ACAATTTCCAGGGTTACCATAGGAATTAACAGGGGATTCATATAAACAAAACAGAAGTTGGAATTGCAAAAAAATAGT-- 3100

Majority --TGTATACTAATAACTGCAT---GCTCAGACA---------AAA-----------------TCTGCCAAA--ATTTGGC

---------+---------+---------+---------+---------+---------+---------+---------+

4650 4660 4670 4680 4690 4700 4710 4720

---------+---------+---------+---------+---------+---------+---------+---------+

TA(AA) --TGTATACTAATAACTGCAT---GCTCAGACA---------AA------------------TCTGCCAAA--ATTTGGC 3134

TA(BB) --TGCATACTAAAAACTGCAT---GGTCAGACA---------AAA-----------------TCTGCCAAA--ATTTGGC 2861

AT --TGTATACTAACAACTGCAT---GCTCAGACA---------AA------------------TCTGCCAAA--ATTTGGC 2724

HV --TGTATACTAAAAATGGCAT---GCTCAGACATGTTCAGACAAA-----------------TCTGCCAAA--ATTTGGC 2962

TU --TGTATACTAATAACTGCAT---GCTCAGACA---------AA------------------TCTGCCAAA--AATTGGC 3138

TS --TGCATACTAAAAACTGCAT---GGTCAGACA---------AAA-----------------TCTGCCAAA--ATTTGGC 2697

BD --TTTCCAGTTTTGTCTGCAT---GGCCAGAGAAATT--G-CCAA-----------------TTTGCAACATCAATTCAC 3459

SB CACTTCTGCCATTGTTAGAACCGAATCTGCTCA--CCATTAACAACTATACACTCCATGCCACATTAAAGAGAGTTTGAG 4577

OS --TGTCTTGTAGTGTCGCCATAAAGTCTGAATATGTCTTTGAGAGCAATCGACCTCTCATATTTCAGCATGAAACTTATG 3178

Majority AACTACATAGTCCTGATGGTATGTT-----TGTTCATCTT-------------------------------------TGT

---------+---------+---------+---------+---------+---------+---------+---------+

4730 4740 4750 4760 4770 4780 4790 4800

---------+---------+---------+---------+---------+---------+---------+---------+

TA(AA) AACTACATAGTCCTGATGGTATGTT-----TGTTCATCTT-------------------------------------TGT 3172

TA(BB) AACCACAAAGTCCTGATGGTACGTT-----TGTTCATCTT-------------------------------------TGT 2899

AT AGCTGCAAAGTCCTGATGGTATGTT-----TGTTCATCTT-------------------------------------TGT 2762

HV AACTACATAGTCCTGATGGTATGTG-----TGTTCATCTTCTCTTTTTTTGTGTGACATTGAATGTGTGTTCACCTTTGT 3037

TU AACTACATAGTCCTGATGGTATGTT-----TGTTCATCTT-------------------------------------TGT 3176

TS AACTACAAAGTCCTGATGGTATGTT-----TGTTCATCTT-------------------------------------TGT 2735

BD GAGTTAATTTTTCTGATAATGT----------TTCATCTT-------------------------------------TAT 3492

SB CATTCTGTGGTACTTACACTGA--CAACCTAGCCCATGGCTGTAGAGGATATGTCTTTTGTGCCCACACCCAACGGGTGT 4655

OS CAGTATCATCTATCAACTTTGTTTTCAGCCTTTTCAGCTTCCCTATACTACGGATGTGAC-AATCTATGTTTGCTCTCTT 3257

Majority CCA-GGTACTTGT-----ATATAAGGTGGGGGG---TAGAGCTGTCGAACAT-----GACGGTGGTTGTTGGTGAAAGCG

---------+---------+---------+---------+---------+---------+---------+---------+

4810 4820 4830 4840 4850 4860 4870 4880

---------+---------+---------+---------+---------+---------+---------+---------+

TA(AA) CCA-GGTACTTGT-----ATATAAGGTGGGGCG---TAGAGCTGTCGAACAT-----GACGGTGGTTGTTGGTGAAAGCG 3238

TA(BB) GCA-GGTACTTGT-----ATATAAGGTGGGGTG---TAGAGCTGTCGAACAT-----GACGGTGGTTGTCGGTGAAAGCG 2965

AT CCA-GGTACTTGT-----ATATAAGGTGGGGGG---TAGAGCTGTAGAACAT-----GACGGTGGTTGTCGAAGAAAGCG 2828

HV CCA-GGTACATGT-----ATATAAGGTGGGGGGG--TAGAGCTGTCAAAGAT-----GACGGTGGTTGTTGGAGAAAGCG 3104

TU CCA-GGTACTTGT-----ATATAAGGTGGGGCG---TAGAGCTGTCGAACAT-----GACGGTGGTTGTTGGTGAAAGCG 3242

TS CCA-GGTACTTGT-----ATATAAGGTGGGGGG---TAGAGCTGTCAAACAT-----GACGGTGGTTGTTGGTGAAAGCG 2801

BD CCA-GGTACTTGT-----ATATAAGATGGGGGG---TGGAACTGTCAAACAT-----GACAGTGGTTGTTGGTGAAAGTG 3558

SB GTAAGGCATATCCCACCAGTTCAAGATTATGAATACCAAGGCCACCGAATTCCAGTGGACACTGCACTCTTTCGCAGGGC 4735

OS GTA-GGTACTTGT-----ATATAAGATGGGGGG---TAGAGCTGTCAAATAT-----GACAGTGGTTGTTGGTGAAAGTG 3323

Majority GCGATACAG--ATTATGAAGGGCTACTCGGAGGCGT----GCAGAAGACCATCATACTCAA-----AGGCTCATTTAATT

---------+---------+---------+---------+---------+---------+---------+---------+

4890 4900 4910 4920 4930 4940 4950 4960

---------+---------+---------+---------+---------+---------+---------+---------+

TA(AA) GCGATACAG--ATTATGAAGGGCTACTCGGAGGCGT----GCAGAAGACCATCATACTCAA-----AGGCTCATTTAATT 3307

TA(BB) GCGATACAG--ATTATGAAGGGCTGCTCGGAGGCGT----GCAGAAGACCATCGTACTCAA-----AGGCTCGTTTAATT 3034

AT GCGATACAG--TTTATGAAGGGCTACTCGGAGGTGT----GCAGAAGACCATCATACTCAA-----AGGTTCATTTAATT 2897

HV GCGATACAG--ATTATGAAGGGCTACGAGGAGGGAT----GCAGAAGACCATCATACTCAA-----AGGCTCATCTAATT 3173

TU GCGATACAG--ATTATGAAGGGCTACTCGGAGGCGT----GCAGAAGACCATCATACTCAA-----AGGCTCATTTAATT 3311

TS GCGATACAG--ATTATGAAGGGCTACTCGGAGGCGT----GCAGAAGACCATCATACTCAA-----AGGCTCATTTAATT 2870

BD GTGATACAG--ATTATGATGGACTACTTGGAGGCGT----ACACAAGACCATCATACTGAA-----AGGCTCATTTAATG 3627

SB ATCAGACAATTACCTCCATTGGCTAGTTCTTGGCCCTTCCATAGAGAGTCTCTGCGGCTGAGACTGAGGCTGATGTAGTA 4815

OS GTGATACTG--ATTATGAAGGACTATTAGGAGGCGT----GCACAAGACCATTATTCTCAA-----AGGCTCATTTAATG 3392

Majority CXGCGCCAAACCAGCTTCATGCCGCCAGAAGCTATTCGCTAGAGGATGTCGTATCGTTTGACAAGCCAGGAATTGCTTCC

---------+---------+---------+---------+---------+---------+---------+---------+

4970 4980 4990 5000 5010 5020 5030 5040

---------+---------+---------+---------+---------+---------+---------+---------+

TA(AA) CCGCGCCAAACCAGCTTCATGCCGCCAGAAACTATTCGCTAGAGGATGTCGTATCGTTTGACAAGCCAGGAATTGCTTCC 3387

TA(BB) CTGCGCCAAACCAGCTTCATGCCGCCAGAAGCTATTCGCTAGAGGATGTCGTATCGTTCGACAAGCCAGGAATTGCTTCC 3114

AT CGGCTCCAAACCACCTTCATGCCGCCAGAAGCTATTCGCTAGAGGATGTCGTATCGTTTGACAAGCCAGGAATTGCTTCT 2977

HV CCGTGCCAAACCAGCGTCATGCCGCCAGAAGCTATACGCGAGAGGATGTTGTATCGTTCGACAAGCCAGGAACCGCGAGC 3253

TU CCGCGCCAAACCAGCTTCATGCCGCCAGAAACTATTCGCTAGAGGATGTCGTATCGTTTGACAAGCCAGGAATTGCTTCC 3391

TS CCGCGCCAAACCAGCTTCATGCCGCCAGAAGCTATTCGCTAGAGGATGTCGTATCTTTCGACAAGCCAGGAATTGCTTCC 2950

BD CTGCTCCAAACCAACTTCATGCGGCCAGAGGCTATTCATTAGAGGATGTTGTATCGTTTGATAAACCAGGAATTTCTTCA 3707

SB CTGAGGACATTCGAGACTGATTATCTGAAGAATCGTCATTCAGAGAACTGGTTCACACTGTCAGCTGAGCCACTGCTTGA 4895

OS CTGTTCCTAACCAAGTCCATGCTGCCAGAAGTTATTCATTACAGGATGTCATATCCTTTGACAAACCAGGAATTACTTCA 3472

Majority GTCGAXGGTTATGCCCCAGATAXCCTAAAATCAGCTCTACAACAATTTGGTGCCCTGGAGGGCXXXXXXXXXXXXXXXXX

---------+---------+---------+---------+---------+---------+---------+---------+

5050 5060 5070 5080 5090 5100 5110 5120

---------+---------+---------+---------+---------+---------+---------+---------+

TA(AA) GTCGACGGTTATGCCCCAGATATCCTAAAATCAGCTCTACAACAATTTGGTGCCCTGCAGGGC 3450

TA(BB) GTCGACGGTTATGCCCCAGATAACCTGAAATCAGCTCTACAACAATTTGGTGCCCTGGAGGGC 3177

AT GTCGACGGTTACGCCCCAGATATCCTAAAATCAGCTCTACAACAATTTGGTGCCCTGGAGGGC 3040

HV GTCGAGGGGTACGCCCCAAATAACCTAAAATCCGCCCTACAACAATTGGGCGCCCGGGAGGGC 3316

TU GTCGACGGTTATGCCCCAGATATCCTAAAATCAGCTCTACAACAATTTGGTGCCCTGCAGGGC 3454

TS GTCGAGGGTTACGCCCCAGATATCCTAAAATCAGCTCTACAACAATTTGGTGCCCTGCAGGGC 3013

BD GTTGAGGGATATCTCCCAGATGACCTAAAATCAGCTCTACAACAATTTGGTGTACTGAAAGACTAAACACTGATTATGGG 3787

SB CCTG----TTGCCTCCTGCTTTGCCAAGAGTT-GTTGCTCAACCGCGTATTGCTCCAGAGTCTTCATAGTATGATTTACT 4970

OS ATCGAGGGATATGGTCCAGATAACCTTAAGTCAGCTCTACAACAATTTGGTATACTGAAAGACAATGTTTAA 3544

Majority XXXXXXXXXXXXXXXXXXXXXXXXXXXXXXXXXXXXXXXXXXXXXXXXXXXXXXXXXXXXXXXXXXXXXXXXXXXXXXXX

---------+---------+---------+---------+---------+---------+---------+---------+

5130 5140 5150 5160 5170 5180 5190 5200

---------+---------+---------+---------+---------+---------+---------+---------+

TA(AA) 3450

TA(BB) 3177

AT 3040

HV 3316

TU 3454

TS 3013

BD TAACCGTGATGTTATTGATGAAATGTCAAGCACAAATGGAGCTGTTTTGACATCAGTACTCAAGTTGATTGACAAATACG 3867

SB TTGTCCTGAAGTTCTTGCTGTGCTGT-GCCAATGTTGAAGACTCGAGCAAAGAGAAGATTCAAACTGTCGAGGATGTGCT 5049

OS 3544

Majority XXXXXXXXXXXXXXXXXXXXXXXXXXXXXXXXXXXXXXXXXXXXXXXXXXXXXXXXXXXXXXXXXXXXXXXXXXXXXXXX

---------+---------+---------+---------+---------+---------+---------+---------+

5210 5220 5230 5240 5250 5260 5270 5280

---------+---------+---------+---------+---------+---------+---------+---------+

TA(AA) 3450

TA(BB) 3177

AT 3040

HV 3316

TU 3454

TS 3013

BD ATCTATATGGTCAAGTGGCATACCCCAAGCACCATAAGCAATCTGATGTCCCAGATATTTACCGTTTG---GCAGCAAGA 3944

SB CCCAACATTCCTCATGATGACGCTCTGAACACTCTTGTTCAGACATTGCGTCGAGCATGAAGATCGTGTGTGCTGAAGGT 5129

OS 3544

Majority XXXXXXXXXXXXXXXXXXXXXXXXXXXXXXXXXXXXXXXXXXXXXXXXXXXXXXXXXXXXXXXXXXXXXXXXXXXXXXXX

---------+---------+---------+---------+---------+---------+---------+---------+

5290 5300 5310 5320 5330 5340 5350 5360

---------+---------+---------+---------+---------+---------+---------+---------+

TA(AA) 3450

TA(BB) 3177

AT 3040

HV 3316

TU 3454

TS 3013

BD ACCAAGGTAACCGAGTCTTATATCTTCATATTTTGAAATATGTGAAAATAGAGTTCTGCAATAAGCATGCTACCGAATTC 4024

SB TCAGGGGGAGCCATGATGGACTGACTGGAAGCTGACCAGAGGTGGAGTGGAATTGCAGGGTCGATTTCCTCATTAAGGTA 5209

OS 3544

Majority XXXXXXXXXXXXXXXXXXXXXXXXXXXXXXXXXXXXXXXXXXXXXXXXXXXXXXXXXXXXXXXXXXXXXXXXXXXXXXXX

---------+---------+---------+---------+---------+---------+---------+---------+

5370 5380 5390 5400 5410 5420 5430 5440

---------+---------+---------+---------+---------+---------+---------+---------+

TA(AA) 3450

TA(BB) 3177

AT 3040

HV 3316

TU 3454

TS 3013

BD TCTGTGGTACCATGATTAATCGTTAATTGGATGTTATAGATTAAGTGGTAATTAAGTTTTAACCTGTCTGTATTTATTA 4103

SB CGAAGGATATCAAGATGGGCAAACCAACTGCATTTCTCAGATCAAGGCCAACTGGATTTCAGAATCCAGACACGAAATGC 5289

OS 3544

Majority XXXXXXXXXXXXXXXXXXXXXXXXXXXXXXXXXXXXXXXXXXXXXXXXXXXXXXXXXXXXXXXXXXXXXXXXXXXXXXXX

---------+---------+---------+---------+---------+---------+---------+---------+

5450 5460 5470 5480 5490 5500 5510 5520

---------+---------+---------+---------+---------+---------+---------+---------+

TA(AA) 3450

TA(BB) 3177

AT 3040

HV 3316

TU 3454

TS 3013

BD 4103

SB GCCCGGAACCGAGTGAGAAACAGCGCAGTGCGTTGAACCGAGAAGAATCCCCAACACCCACACTGCCACCCAGCAGACCA 5369

OS 3544

Majority XXXXXXXXXXXXXXXXXXXXXXXXXXXXXXXXXXXXXXXXXXXXXXXXXXXXXXXXXXXXXXXXXXXXXXXXXXXXXXXX

---------+---------+---------+---------+---------+---------+---------+---------+

5530 5540 5550 5560 5570 5580 5590 5600

---------+---------+---------+---------+---------+---------+---------+---------+

TA(AA) 3450

TA(BB) 3177

AT 3040

HV 3316

TU 3454

TS 3013

BD 4103

SB AGAATACTGCCACCCACGCTGCCAGAATCCTGGTCTACCGTCGCCTGTGCAGCCAAAATTAATCCACCGCCATCTTCACT 5449

OS 3544

Majority XXXXXXXXXXXXXXXXXXXXXXXXXXXXXXXXXXXXXXXXXXXXXXXXXXXXXXXXXXXXXXXXXXXXXXXXXXXXXXXX

---------+---------+---------+---------+---------+---------+---------+---------+

5610 5620 5630 5640 5650 5660 5670 5680

---------+---------+---------+---------+---------+---------+---------+---------+

TA(AA) 3450

TA(BB) 3177

AT 3040

HV 3316

TU 3454

TS 3013

BD 4103

SB GCCAAATCCCCATAACTCGTGTCGTCAGGACCAAGATGACCGAGGATATGTCACCTCTTGATTACCAGCTATCACACCCT 5529

OS 3544

Majority XXXXXXXXXXXXXXXXXXXXXXXXXXXXXXXXXXXXXXXXXXXXXXXXXXXXXXXXXXXXXXXXXXXXXXXXXXXXXXXX

---------+---------+---------+---------+---------+---------+---------+---------+

5690 5700 5710 5720 5730 5740 5750 5760

---------+---------+---------+---------+---------+---------+---------+---------+

TA(AA) 3450

TA(BB) 3177

AT 3040

HV 3316

TU 3454

TS 3013

BD 4103

SB TGGCCGCCGCCAATGTTCTGGCAACATGAGCTAGAACAACCAGATCGAGATACGAGGAATGTGACATCTCCTTTACCTGT 5609

OS 3544

Majority XXXXXXXXXXXXXXXXXXXXXXXXXXXXXXXXXXXXXXXXXXXXXXXXXXXXXXXXXXXXXXXXXXXXXXXXXXXXXXXX

---------+---------+---------+---------+---------+---------+---------+---------+

5770 5780 5790 5800 5810 5820 5830 5840

---------+---------+---------+---------+---------+---------+---------+---------+

TA(AA) 3450

TA(BB) 3177

AT 3040

HV 3316

TU 3454

TS 3013

BD 4103

SB GAAATTGTTCACCTTCCTTACGGACGCCTGGCCTTTAAGCACTCGCACCAACATTGCACATTAGGTCCTCACAACCAATG 5689

OS 3544

Majority XXXXXXXXXXXXXXXXXXXXXXXXXXXXXXXXXXXXXXXXXXXXXXXXXXXXXXXXXXXXXXXXXXXXXXXXXXXXXXXX

---------+---------+---------+---------+---------+---------+---------+---------+

5850 5860 5870 5880 5890 5900 5910 5920

---------+---------+---------+---------+---------+---------+---------+---------+

TA(AA) 3450

TA(BB) 3177

AT 3040

HV 3316

TU 3454

TS 3013

BD 4103

SB AAACATCCACACAAGTCTGATACACGCGCACAGAGACGCTGTTGATTCCCATTGCTAGTTCTTGGTCCTGAGCGACCCCC 5769

OS 3544

Majority XXXXXXXXXXXXXXXXXXXXXXXXXXXXXXXXXXXXXXXXXXXXXXXXXXXXXXXXXXXXXXXXXXXXXXXXXXXXXXXX

---------+---------+---------+---------+---------+---------+---------+---------+

5930 5940 5950 5960 5970 5980 5990 6000

---------+---------+---------+---------+---------+---------+---------+---------+

TA(AA) 3450

TA(BB) 3177

AT 3040

HV 3316

TU 3454

TS 3013

BD 4103

SB TGGCGTGACACCAAAATGCCTTATCATTCAGTGACTACGTGTAATAAACTCTTCTATACTGAATGCAGACTGGTGAAAAC 5849

OS 3544

Majority XXXXXXXXXXXXXXXXXXXXXXXXXXXXXXXXXXXXXXXXXXXXXXXXXXXXXXXXXXXXXXXXXXXXXXXXXXXXXXXX

---------+---------+---------+---------+---------+---------+---------+---------+

6010 6020 6030 6040 6050 6060 6070 6080

---------+---------+---------+---------+---------+---------+---------+---------+

TA(AA) 3450

TA(BB) 3177

AT 3040

HV 3316

TU 3454

TS 3013

BD 4103

SB TCAACTTGGTGTTATCGTTTCCTGTTTCCATCCTTTAATAGGTGTATCTGTGCTGTACATTATAATGTTATTTTCAATGA 5929

OS 3544

Majority XXXXXXXXXXXXXXXXXXXXXXXXXXXXXXXXXXXXXXXXXXXXXXXXXXXXXXXXXXXXXXXXXXXXXXXXXXXXXXXX

---------+---------+---------+---------+---------+---------+---------+---------+

6090 6100 6110 6120 6130 6140 6150 6160

---------+---------+---------+---------+---------+---------+---------+---------+

TA(AA) 3450

TA(BB) 3177

AT 3040

HV 3316

TU 3454

TS 3013

BD 4103

SB TACATATCGCGTGAAAAAAATCTTAAGGCCTTGTTTGGATGTAGTCGGATTCACATCAATCCACATGTATTGAGGTGGAT 6009

OS 3544

Majority XXXXXXXXXXXXXXXXXXXXXXXXXXXXXXXXXXXXXXXXXXXXXXXXXXXXXXXXXXXXXXXXXXXXXXXXXXXXXXXX

---------+---------+---------+---------+---------+---------+---------+---------+

6170 6180 6190 6200 6210 6220 6230 6240

---------+---------+---------+---------+---------+---------+---------+---------+

TA(AA) 3450

TA(BB) 3177

AT 3040

HV 3316

TU 3454

TS 3013

BD 4103

SB TGAAGTGAAATTTGAACTAAATTCCACCTTAATCCACACCAACACACATGGATTGAAGTGAATCCGACAACATCCAAACA 6089

OS 3544

Majority XXXXXXXXXXXXXXXXXXXXXXXXXXXXXXXXXXXXXXXXXXXXXXXXXXXXXXXXXXXXXXXXXXXXXXXXXXXXXXXX

---------+---------+---------+---------+---------+---------+---------+---------+

6250 6260 6270 6280 6290 6300 6310 6320

---------+---------+---------+---------+---------+---------+---------+---------+

TA(AA) 3450

TA(BB) 3177

AT 3040

HV 3316

TU 3454

TS 3013

BD 4103

SB AGGCCTAAAGTGGGAGCATGTAAGATTTATGGAACATGTTAGTCTATATGTGGTGTATGGACTGCTGGAGATGTATGGTG 6169

OS 3544

Majority XXXXXXXXXXXXXXXXXXXXXXXXXXXXXXXXXXXXXXXXXXXXXXXXXXXXXXXXXXXXXXXXXXXXXXXXXXXXXXXX

---------+---------+---------+---------+---------+---------+---------+---------+

6330 6340 6350 6360 6370 6380 6390 6400

---------+---------+---------+---------+---------+---------+---------+---------+

TA(AA) 3450

TA(BB) 3177

AT 3040

HV 3316

TU 3454

TS 3013

BD 4103

SB GTTACGCACATAAACAAAGGTCAAAAGAGATGTAGAAAGTACCAATATATTTGCTATCTAGTCTCATTTAAAATAATCAT 6249

OS 3544

Majority XXXXXXXXXXXXXXXXXXXXXXXXXXXXXXXXXXXXXXXXXXXXXXXXXXXXXXXXXXXXXXXXXXXXXXXXXXXXXXXX

---------+---------+---------+---------+---------+---------+---------+---------+

6410 6420 6430 6440 6450 6460 6470 6480

---------+---------+---------+---------+---------+---------+---------+---------+

TA(AA) 3450

TA(BB) 3177

AT 3040

HV 3316

TU 3454

TS 3013

BD 4103

SB TTTGCAATTCTTAATTTATGACGTAATTCTCCACCAGAATGTCTGAAACAGCCTTTTGAGCAATGTAACCTTCACATATT 6329

OS 3544

Majority XXXXXXXXXXXXXXXXXXXXXXXXXXXXXXXXXXXXXXXXXXXXXXXXXXXXXXXXXXXXXXXXXXXXXXXXXXXXXXXX

---------+---------+---------+---------+---------+---------+---------+---------+

6490 6500 6510 6520 6530 6540 6550 6560

---------+---------+---------+---------+---------+---------+---------+---------+

TA(AA) 3450

TA(BB) 3177

AT 3040

HV 3316

TU 3454

TS 3013

BD 4103

SB CACATTTTTGTAGAATAAACTTTATCCCTGTATCTGTGTGTTTTTTCAGGGTGAGAAAAATTCCGAACAACAATTTTCTG 6409

OS 3544

Majority XXXXXXXXXXXXXXXXXXXXXXXXXXXXXXXXXXXXXXXXXXXXXXXXXXXXXXXXXXXXXXXXXXXXXXXXXXXXXXXX

---------+---------+---------+---------+---------+---------+---------+---------+

6570 6580 6590 6600 6610 6620 6630 6640

---------+---------+---------+---------+---------+---------+---------+---------+

TA(AA) 3450

TA(BB) 3177

AT 3040

HV 3316

TU 3454

TS 3013

BD 4103

SB ATGGTGTTTGATCATTCGTTGATCATTTTTGCAGGTATTTGTATATCAGATGGGGGGTAGAGCTGTCAAACATCACTGTG 6489

OS 3544

Majority XXXXXXXXXXXXXXXXXXXXXXXXXXXXXXXXXXXXXXXXXXXXXXXXXXXXXXXXXXXXXXXXXXXXXXXXXXXXXXXX

---------+---------+---------+---------+---------+---------+---------+---------+

6650 6660 6670 6680 6690 6700 6710 6720

---------+---------+---------+---------+---------+---------+---------+---------+

TA(AA) 3450

TA(BB) 3177

AT 3040

HV 3316

TU 3454

TS 3013

BD 4103

SB GTTGTCGGTGAATGTGGTGACACAGATTATGAAGGACTACTTGGAGGCGTGCACAAAACTATCATACTCAAAGGCTCGTT 6569

OS 3544

Majority XXXXXXXXXXXXXXXXXXXXXXXXXXXXXXXXXXXXXXXXXXXXXXXXXXXXXXXXXXXXXXXXXXXXXXXXXXXXXXXX

---------+---------+---------+---------+---------+---------+---------+---------+

6730 6740 6750 6760 6770 6780 6790 6800

---------+---------+---------+---------+---------+---------+---------+---------+

TA(AA) 3450

TA(BB) 3177

AT 3040

HV 3316

TU 3454

TS 3013

BD 4103

SB CAATACTGCTCCAAACCAAGTCCATGCTAACAGAAGCTATTCATTCCAAGATGTTGTATCCCTTGAGAAACAAGGAATTG 6649

OS 3544

Majority XXXXXXXXXXXXXXXXXXXXXXXXXXXXXXXXXXXXXXXXXXXXXXXXXXXXXXXXXXXXXXXXXXXXXXXXXXXXXXXX

---------+---------+---------+---------+---------+---------+---------+---------+

6810 6820 6830 6840 6850 6860 6870 6880

---------+---------+---------+---------+---------+---------+---------+---------+

TA(AA) 3450

TA(BB) 3177

AT 3040

HV 3316

TU 3454

TS 3013

BD 4103

SB CTTCAATTGAGGGATATGGTCCAGACAATCTAAAATCAGCTCTAAGGCAATTTGGTATATCGAAAGACTAAATCTTTGAT 6729

OS 3544

Majority XXXXXXXXXXXXXXXXXXXXXXXXXXXXX

---------+---------+---------

6890 6900

---------+---------+---------

TA(AA) 3450

TA(BB) 3177

AT 3040

HV 3316

TU 3454

TS 3013

BD 4103

SB CTTGCTGGTCAGCAGAGGAATCAAACTAG 6758

OS 3544

Additional file 3

Sequence alignment of SPSII gene region containing six exons and six introns (intron 7 to exon 13) studied in nine genomes (without Triticum aestivum D genome). TA(AA): Triticum aestivum-A genome, TA(BB): Triticum aestivum-B genome, AT: Aegilops tauschii, HV: Hordeum vulgare, TU: Triticum urartu, TS: Triticum speltoides, BD: Brachypodium distachyon, SB: Sorghum bicolor, OS: Oryza sativa. Sequences from 5` UTR and 3´ end are not shown due to unavailbilty of all sequeneces in some cases. Positions with SNP represented by grey boxes. Sequence in red font represented genome specific primers with 3´ end SNP. Sequence in red font with underline represented overlapping forward and reverse genome specific primers with 3´ end SNP. Unspliced intron in TA (BB) sequence is shown by bold italic font. TA(AA): Triticum aestivum A genome, TA(BB): Triticum aestivum B genome, TU: Triticum urartu, TS: Triticum speltoides, AT: Aegilops tauschii, HV: Hordeum vulgare, OS: Oryza sativa, SB: Sorghum bicolor, BD: Brachypodium distachyon.
