# Supplementary material for: Delineating the structural, functional and evolutionary relationships of sucrose phosphate synthase gene family II in wheat and related grasses
Source: BMC Plant Biol. 2010 Jun 30;10:134. doi: 10.1186/1471-2229-10-134 (PMC3017794; doi:10.1186/1471-2229-10-134)
Supplement: Additional file 5 — Sequence alignment of six introns (intron 7-12) of SPSII gene that could be studied in nine genomes (without Triticum aestivum D genome) using MegAlign (ClustalW, slow/accurate). Boxes represented residues different from the consensus. TA(AA): Triticum aestivum A genome, TA(BB): Triticum aestivum B genome, TU: Triticum urartu, TS: Triticum speltoides, AT: Aegilops tauschii, HV: Hordeum vulgare, OS: Oryza sativa, SB: Sorghum bicolor, BD: Brachypodium distachyon. [file 1471-2229-10-134-S5.DOC]

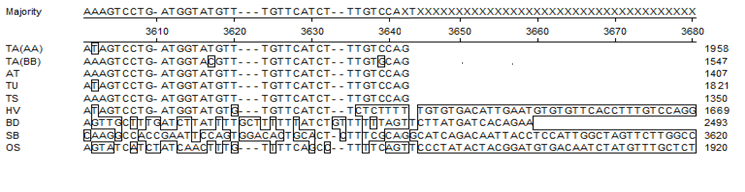


Additional file 5

Sequence alignment of six introns (intron 7-12) of SPSII gene that could be studied in nine genomes (without Triticum aestivum D genome) using MegAlign (ClustalW, slow/accurate). Boxes represented residues different from the consensus. TA(AA): Triticum aestivum A genome, TA(BB): Triticum aestivum B genome, TU: Triticum urartu, TS: Triticum speltoides, AT: Aegilops tauschii, HV: Hordeum vulgare, OS: Oryza sativa, SB: Sorghum bicolor, BD: Brachypodium distachyon.
