# Supplementary material for: Delineating the structural, functional and evolutionary relationships of sucrose phosphate synthase gene family II in wheat and related grasses
Source: BMC Plant Biol. 2010 Jun 30;10:134. doi: 10.1186/1471-2229-10-134 (PMC3017794; doi:10.1186/1471-2229-10-134)
Supplement: Additional file 6 — Sequence similarity and divergence of SPSII gene based on sequence alignment of different regions (a) Sequence similarity and divergence of SPSII gene based on region studied in all ten genomes (including Triticum aestivum D genome). (b) Sequence similarity and divergence of SPSII gene based on six exons (exon 8-13) compared in nine genomes (without Triticum aestivum D genome). (c) Sequence similarity and divergence of SPSII gene based on six introns (intron 7-12) compared in nine genomes (without Triticum aestivum D genome). TA(AA): Triticum aestivum A genome, TA(BB): Triticum aestivum B genome, TU: Triticum urartu, TS: Triticum speltoides, AT: Aegilops tauschii, HV: Hordeum vulgare, OS: Oryza sativa, SB: Sorghum bicolor, BD: Brachypodium distachyon. [file 1471-2229-10-134-S6.DOC]

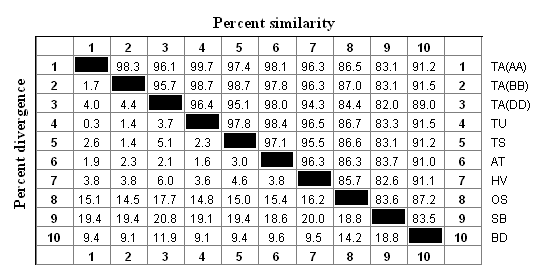


(a)


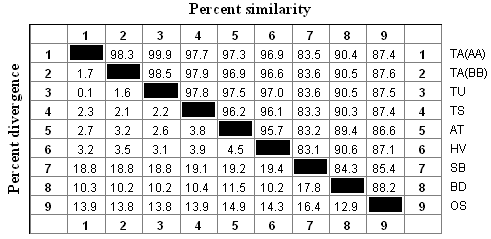


(b)


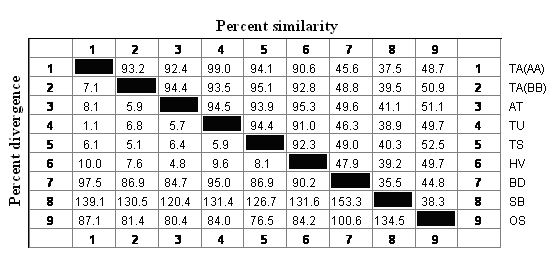


(c)

Additional file 6

Sequence similarity and divergence of SPSII gene based on sequence alignment of different regions (a) Sequence similarity and divergence of SPSII gene based on region studied in all ten genomes (including Triticum aestivum D genome). (b) Sequence similarity and divergence of SPSII gene based on six exons (exon 8-13) compared in nine genomes (without Triticum aestivum D genome). (c) Sequence similarity and divergence of SPSII gene based on six introns (intron 7-12) compared in nine genomes (without Triticum aestivum D genome). TA(AA): Triticum aestivum A genome, TA(BB): Triticum aestivum B genome, TU: Triticum urartu, TS: Triticum speltoides, AT: Aegilops tauschii, HV: Hordeum vulgare, OS: Oryza sativa, SB: Sorghum bicolor, BD: Brachypodium distachyon.
